# Supplementary material for: Incidentally diagnosed cancer and commonly preceding clinical scenarios: a cross-sectional descriptive analysis of English audit data
Source: BMJ Open. 2019 Sep 17;9(9):e028362. doi: 10.1136/bmjopen-2018-028362 (PMC6756358; doi:10.1136/bmjopen-2018-028362)

Supplementary files for Koo et al., 2019

Supplementary information for "*Incidentally diagnosed cancer and commonly preceding clinical scenarios: a cross-sectional descriptive analysis of English audit data*" Koo et al., 2019

Table of contents

- Supplementary Figure 1: Flow chart describing sample derivation for main analysis
- Supplementary Table 1: Cancer site case-mix of incidentally diagnosed cancer patients
- *Sensitivity analysis using a broader definition of incidental diagnosis*
    - Supplementary Table 2: Characteristics of incidental cancer patients versus non-incidental cancer patients, and crude/adjusted odds ratios of incidental status (n=14,082)
    - Supplementary Figure 2: Odds ratios of incidental versus symptomatic diagnosis of cancer by cancer site (n=14,082; reference group: colorectal cancer)

Supplementary files for Koo et al., 2019

Supplementary Figure 1: Flow chart describing sample derivation for main analysis

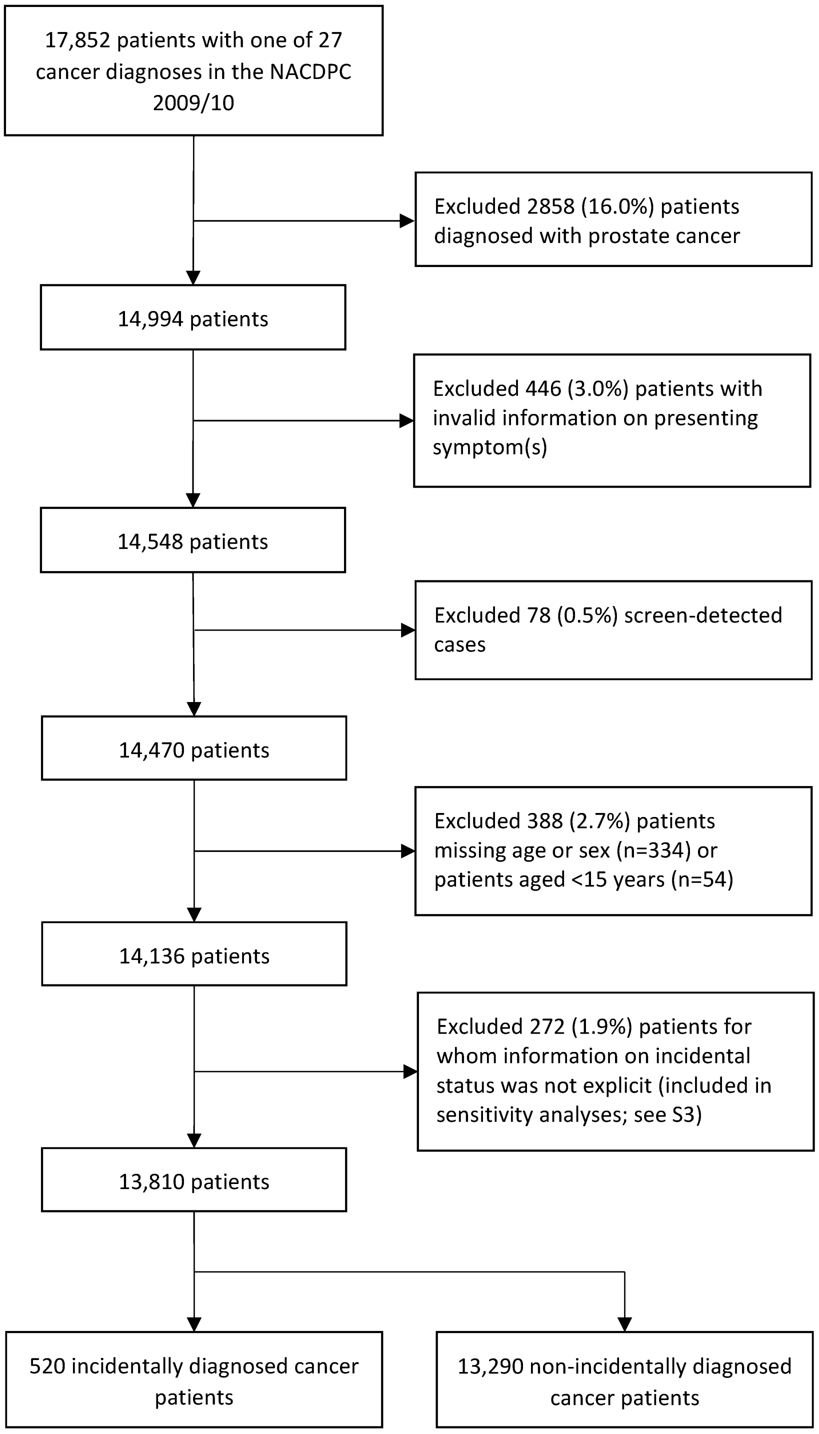

Supplementary files for Koo et al., 2019

Supplementary Table 1: Cancer site case-mix of incidentally diagnosed cancer patients

Cancer	N	% (95% CI)[†]
Leukaemia*	103	20% (17–23%)
Lung	77	15% (12–18%)
Colorectal	66	13% (10–16%)
Renal	46	9% (7–12%)
Breast	34	7% (5–9%)
Lymphoma	33	6% (5–9%)
Melanoma	30	6% (4–8%)
Bladder	28	5% (4–8%)
Myeloma	20	4% (3–6%)
Thyroid	13	3% (1–4%)
Liver	11	2.1% (1.2–3.7%)
Ovarian	10	1.9% (1.0–3.5%)
Stomach	8	1.5% (0.8–3.0%)
Endometrial	6	1.2% (0.5–2.5%)
Pancreatic	6	1.2% (0.5–2.5%)
Gallbladder	5	1.0% (0.4–2.2%)
Oropharyngeal	5	1.0% (0.4–2.2%)
Mesothelioma	4	0.8% (0.3–2.0%)
Laryngeal	3	0.6% (0.2–1.7%)
Oesophageal	3	0.6% (0.2–1.7%)
Vulval	3	0.6% (0.2–1.7%)
Brain	2	0.4% (0.1–1.4%)
Testicular	2	0.4% (0.1–1.4%)
Sarcoma*	1	0.2% (0.03–1.1%)
Small Intestine	1	0.2% (0.03–1.1%)
Total	520	100%

[†] Proportion of patients with each cancer site, of the total incidentally diagnosed population (n=520)

*No information was available on leukaemia or sarcoma type.

Supplementary files for Koo et al., 2019

Supplementary Table 2: Characteristics of incidental cancer patients versus non-incidental cancer patients, and crude/adjusted odds ratios of incidental status (n=14,082)

	Total	Incidental		Crude	Adjusted[a]
	N	n	% (95% CI)	OR (95% CI)	OR (95% CI)
Total	14082	792	6% (5–6%)	-	-
**Sex**				**0.001**[b]	**0.045**[b]
Men	5983	422	7% (6–8%)	Ref.	Ref.
Women	8099	370	5% (4–5%)	0.63 (0.55–0.73)	0.84 (0.71–1.00)
**Age group**				**<0.001**[b]	**<0.001**[b]
15–49 years	2089	48	2% (2–3%)	0.36 (0.26–0.50)	0.36 (0.26–0.51)
50–59 years	2080	95	5% (4–6%)	0.73 (0.57–0.94)	0.75 (0.58–0.98)
60–69 years	3264	200	6% (5–7%)	Ref.	Ref.
70–79 years	3739	253	7% (6–8%)	1.11 (0.92–1.35)	1.08 (0.89–1.33)
80+ years	2910	196	7% (6–8%)	1.11 (0.90–1.36)	1.17 (0.94–1.45)
**Cancer site**				**<0.001**[b]	**<0.001**[b]
Leukaemia*	511	164	32% (28–36%)	11.25 (8.55–14.81)	12.48 (9.46–16.48)
Liver	116	24	21% (14–29%)	6.21 (3.79–10.16)	6.28 (3.82–10.32)
Renal	373	63	17% (13–21%)	4.84 (3.45–6.78)	5.12 (3.64–7.19)
Thyroid	113	16	14% (9–22%)	3.93 (2.23–6.92)	5.93 (3.31–10.60)
Myeloma	241	33	14% (10–19%)	3.78 (2.48–5.74)	3.74 (2.46–5.69)
Gallbladder	68	5	7% (3–16%)	1.89 (0.74–4.80)	2.00 (0.78–5.09)
Bladder	869	55	6% (5–8%)	1.61 (1.15–2.26)	1.52 (1.08–2.14)
Lung	1913	115	6% (5–7%)	1.52 (1.15–2.01)	1.50 (1.14–1.97)
Lymphoma	704	39	6% (4–7%)	1.40 (0.95–2.04)	1.64 (1.12–2.41)
Mesothelioma	75	4	5% (2–13%)	1.34 (0.48–3.75)	1.23 (0.44–3.45)
Melanoma	839	35	4% (3–6%)	1.04 (0.70–1.54)	1.30 (0.88–1.94)
Vulval	73	3	4% (1–11%)	1.02 (0.32–3.30)	1.19 (0.37–3.87)
Colorectal	2431	98	4% (3–5%)	Ref.	Ref.
Ovarian	398	14	4% (2–6%)	0.87 (0.49–1.54)	1.05 (0.59–1.88)
Stomach	303	9	3% (2–6%)	0.73 (0.36–1.46)	0.72 (0.36–1.44)
Breast	2717	76	3% (2–3%)	0.69 (0.51–0.93)	0.91 (0.66–1.25)
Pancreatic	374	10	3% (1–5%)	0.65 (0.34–1.27)	0.66 (0.34–1.28)
Laryngeal	121	3	2% (1–7%)	0.61 (0.19–1.94)	0.62 (0.19–1.98)
Oropharyngeal	213	5	2% (1–5%)	0.57 (0.23–1.42)	0.68 (0.27–1.69)
Endometrial	413	9	2% (1–4%)	0.53 (0.27–1.06)	0.62 (0.31–1.24)
Small Intestine	53	1	2% (–10%)	0.46 (0.06–3.35)	0.48 (0.07–3.55)
Sarcoma*	107	2	2% (1–7%)	0.45 (0.11–1.86)	0.57 (0.14–2.36)
Testicular	149	2	1% (–5%)	0.32 (0.08–1.33)	0.66 (0.16–2.76)
Brain	215	2	1% (–3%)	0.22 (0.05–0.91)	0.25 (0.06–1.02)
Cervical	126	1	1% (–4%)	0.19 (0.03–1.38)	0.29 (0.04–2.09)
Oesophageal	567	4	1% (–2%)	0.17 (0.06–0.46)	0.17 (0.06–0.45)

[a] adjusted for sex, age group, and cancer site
[b] joint Wald test p-value
*No information was available on leukaemia or sarcoma type.

Supplementary files for Koo et al., 2019

Supplementary Figure 2: Odds ratios of incidental versus symptomatic diagnosis of cancer by cancer site (n=14,082; reference group: colorectal cancer)

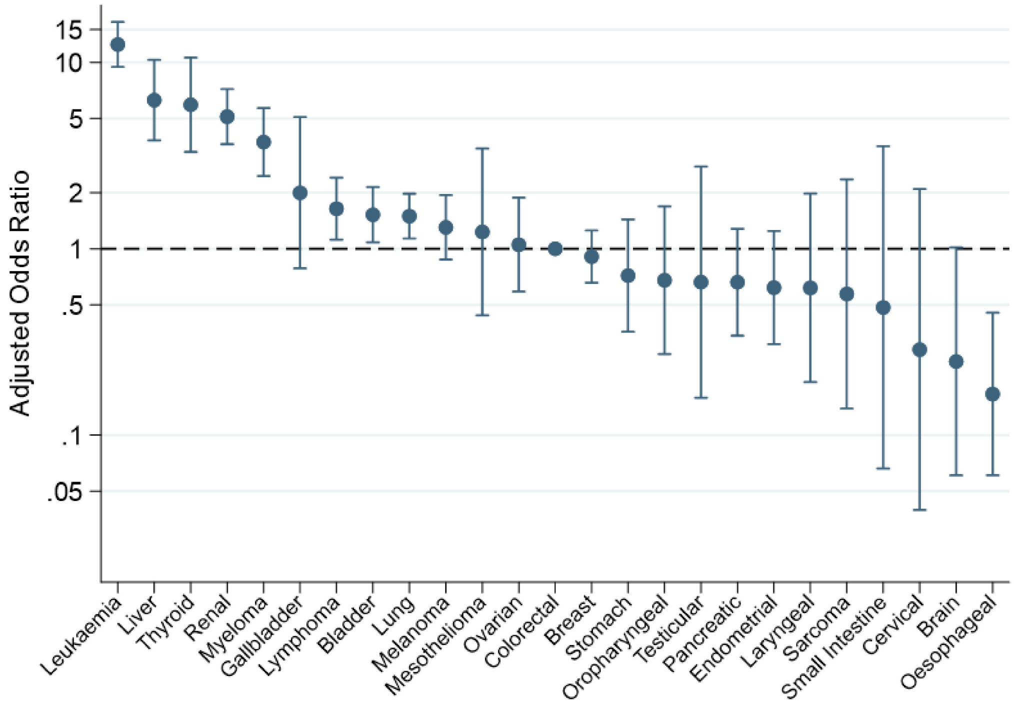

Supplement: Supplementary data [file bmjopen-2018-028362supp001.pdf]
