# Supplementary material for: Incidentally diagnosed cancer and commonly preceding clinical scenarios: a cross-sectional descriptive analysis of English audit data
Source: BMJ Open. 2019 Sep 17;9(9):e028362. doi: 10.1136/bmjopen-2018-028362 (PMC6756358; doi:10.1136/bmjopen-2018-028362)
Supplement: Author's manuscript [file bmjopen-2018-028362.draft_revisions.pdf]

BMJ Open

BMJ Open is committed to open peer review. As part of this commitment we make the peer review history of every article we publish publicly available.

When an article is published we post the peer reviewers' comments and the authors' responses online. We also post the versions of the paper that were used during peer review. These are the versions that the peer review comments apply to.

The versions of the paper that follow are the versions that were submitted during the peer review process. They are not the versions of record or the final published versions. They should not be cited or distributed as the published version of this manuscript.

BMJ Open is an open access journal and the full, final, typeset and author-corrected version of record of the manuscript is available on our site with no access controls, subscription charges or pay-per-view fees (http://bmjopen.bmj.com).

If you have any questions on BMJ Open's open peer review process please email info.bmjopen@bmj.com

BMJ Open

Incidentally diagnosed cancer: population-based evidence on frequency, variation, and commonly preceding clinical scenarios

Journal:	*BMJ Open*
Manuscript ID	bmjopen-2018-028362
Article Type:	Research
Date Submitted by the Author:	04-Dec-2018
Complete List of Authors:	Koo, Minjoung; University College London, Behavioural Science and Health<br>Rubin, Greg; Royal Victoria Infirmary, Institute of Health and Society, Newcastle University<br>McPhail, Sean; Public Health England, National Cancer Registration and Analysis Service; University College London, Behavioural Science and Health<br>Lyratzopoulos, Georgios ; University College London, Department of Behavioural Science and Health; Public Health England, National Cancer Registration and Analysis Service
Keywords:	PRIMARY CARE, RADIOLOGY & IMAGING, PUBLIC HEALTH, HEALTH SERVICES ADMINISTRATION & MANAGEMENT, ONCOLOGY, PATHOLOGY

SCHOLARONE™
Manuscripts

Incidentally diagnosed cancer: population-based evidence on frequency, variation, and commonly preceding clinical scenarios

Minjoung Monica Koo PhD [a], Greg P Rubin MD [b], Sean McPhail PhD [a,c], & Georgios Lyratzopoulos MD [a,c]

Affiliations:
[a] University College London, Gower street, London WC1E 6BT, UK
[b] Institute of Health and Society, Newcastle University, Sir James Spence Institute, Royal Victoria Infirmary, Newcastle upon Tyne, NE1 4LP, UK
[c] National Cancer Registration and Analysis Service, Public Health England, Wellington House, 133-155 Waterloo Road, London, SE1 8UG, UK

Corresponding Author:
Dr Minjoung Monica Koo (monica.koo.14@ucl.ac.uk)

Funding statement:
This work was supported by a grant from the UK Department of Health [grant number no. 106/0001], as part of the program of the Policy Research Unit in Cancer Awareness, Screening and Early Diagnosis. The Policy Research Unit in Cancer Awareness, Screening, and Early Diagnosis receives funding for a research program from the Department of Health Policy Research Programme. It is a collaboration between researchers from seven institutions (Queen Mary University of London, University College London, King's College London, London School of Hygiene and Tropical Medicine, Hull York Medical School, Durham University, and Peninsula Medical School/University of Exeter). GL is supported by a Cancer Research UK Advanced Clinician Scientist Fellowship [grant number: C18081/A18180]. The views expressed are those of the authors and not necessarily those of the Department of Health or Cancer Research UK.

Competing interests:
None declared

Abstract: 264
Main text: 1975
References: 31
Tables: 2
Figures: 3

For peer review only

Abstract

Objectives

Cancer can be diagnosed in the absence of relevant symptoms, but little is known about the frequency and circumstances preceding such diagnoses outside participation in screening programmes. We aimed to examine incidentally diagnosed cancer among a cohort of cancer patients diagnosed in England.

Design

Cross-sectional study of incident cancer patients.

Setting

We analysed free-text information on cancer patients aged 15 or older included in the English National Audit of Cancer Diagnosis in Primary Care (NACDPC) (2009-10). Patients with screen-detected cancers and those diagnosed with prostate cancer were excluded. We examined the odds of incidental cancer diagnosis by patient characteristic and cancer site using logistic regression, and described clinical scenarios leading to incidental diagnosis.

Results

Among the studied cancer patient population (n=13,810), 520 (4%) patients were diagnosed incidentally. The odds of incidental cancer diagnosis increased with age ($p<0.001$), without a difference between men and women after adjustment. Incidental diagnosis was most common among patients with leukaemia (23%), renal (13%) and thyroid cancer (12%), and least common among patients with brain (0.9%), oesophageal (0.5%), and cervical cancer (no cases). Variation in odds of incidental diagnosis by cancer site remained after adjusting for age group and sex.

Incidental diagnoses were commonly preceded by a range of clinical scenarios across primary and secondary care. These included the monitoring or management of pre-existing conditions, routine testing before or after elective surgery, and the investigation of unrelated acute or new conditions.

Conclusions

One in 25 patients with cancer in our population-based cohort were diagnosed incidentally. The epidemiological, clinical, psychological, and economic implications of this diagnostic route merit further investigation.

For peer review only

Strengths and limitations of this study

- The findings are based on a large population-based cohort of individuals diagnosed with a range of cancers
- Diagnostic status (incidental or non-incidental) was identified using free-text information provided by primary care physicians based on primary care records
- We describe common mechanisms of incidental diagnosis beyond a single modality with a high level of detail
- We were unable to examine differences in clinical outcome between incidental and non-incidentally diagnosed cancer patients

For peer review only

INTRODUCTION

Cancer is most often diagnosed following presentation with symptoms caused by the malignancy [1,2]. However, some patients are diagnosed with cancer incidentally, in the absence of symptoms that could not plausibly be related to the tumour and outside formal cancer screening or surveillance activity. The use of imaging technologies (including x-ray, CT, MRI, and PET scans) is one of the commonly described routes to incidental diagnosis of different diseases, including cancer [3–6]. Chronic disease management involving periodic routine blood or urine testing may represent another common pathway to incidental diagnosis and are increasingly used in primary care [7–10]. Nonetheless, evidence regarding the frequency of such incidental diagnoses is currently limited.

Since incidental diagnoses are characterised by the absence of tumour related symptoms, it is plausible that some patients with incidentally detected cancer could be overdiagnosed, whereby the detected cancer would not have otherwise caused symptoms in the patient's lifetime [11]. Concerns about overdiagnosis thus far have largely focused on screening-detected cancers (e.g. breast cancer), but it may be also occurring in other contexts [12,13]. Empirical evidence about the frequency and predictors of incidental diagnosis of cancer is needed alongside the consideration of potential overdiagnosis and subsequent clinical, psychological, or economic consequences of this phenomenon.

We therefore aimed to examine the frequency of incidental diagnosis among an incident cohort of cancer patients; identify patient groups at higher risk of incidental diagnosis; and examine common pathways and mechanisms likely to lead to incidental diagnosis of cancer.

For peer review only

METHODS

Study design and population

We analysed cross-sectional data collected as part of the English National Audit of Cancer Diagnosis in Primary Care (NACDPC) [14]. Briefly, health professionals from 1,170 participating general practices (representing 14% of practices in England) provided information on the diagnostic pathway for a consecutive sample of patients diagnosed with cancer during April 2009–2010. Participating practices were comparable to non-participating practices in (former) respective Cancer Networks, and the patient population was broadly representative of the contemporary national incident cancer patient cohort [14,15]. Clinicians participating in the NACDPC provided information regarding the main presenting symptoms, cancer diagnosis, demographic characteristics, and route of diagnosis based on primary care records.

Definition and identification of cases

Informed by previous literature, we defined the incidental diagnosis of cancer as the diagnosis of cancer in individuals who were either asymptomatic (and not participating in population-based screening programmes), or if symptomatic, with presenting symptoms that could not plausibly be related to their subsequent diagnosis [5,16,17].

The nature of cancer diagnosis (incidental or non-incidental), was ascertained by examination of the free-text information included in the presenting symptoms data field (answering the audit question "what were the main presenting symptom [of the patient]?"). We identified 520 cases where there was an explicit mention of the incidental nature of diagnosis (e.g. by use of phrases including "accidental finding"; "chance finding"; "incidental"; "opportunistic" or other details regarding circumstances indicating an incidental diagnosis).

Patients diagnosed with prostate cancer were excluded *a priori*, given the difficulties in reliably distinguishing reasons for Prostate Specific Antigen testing [18]. Patients with screen-detected breast, colorectal, and cervical cancer, and those diagnosed through surveillance for pre-malignant or high-risk conditions were also excluded. Therefore, the study population comprised 13,810 patients aged 15 or older with sufficient information to determine incidental/non-incidental status, and complete information on cancer diagnosis, age group, and sex (see Figure S1 for sample derivation).

Data analysis

Firstly, we compared the demographic and clinical characteristics of incidentally and non-incidentally diagnosed patients. Logistic regression was used to calculate crude and adjusted odds ratios of incidental diagnosis by sex, age group, and cancer site. We also examined the 'cancer site signature' of the incidentally diagnosed population, i.e. the relatively frequency of each cancer site among incidentally diagnosed patients. Colorectal cancer was used as the reference category for cancer site, as the most common non-gender specific cancer in our population. All statistical analyses were conducted in STATA SE v.15 (StataCorp, College Station, TX, USA).

Subsequently, we identified common clinical scenarios leading to incidental diagnosis based on a subgroup of patients with relevant information (n=345, 66% of all incidental diagnoses). These findings were synthesised narratively.

For peer review only

Sensitivity analysis

The definition of incidentally diagnosed cancer used in the main analysis was based on explicit reference to the incidental nature of cancer diagnosis (see dark blue shaded area in Figure 1). We performed sensitivity analyses expanding the definition of incidental cancer to include an additional 272 patients who were described as having had no symptoms (not otherwise specified), or an abnormal clinical findings (not otherwise specified), or both no symptoms and abnormal clinical findings to the same audit question “what were the main presenting symptom [of the patient]?” (indicated by the light blue shaded area in Figure 1).

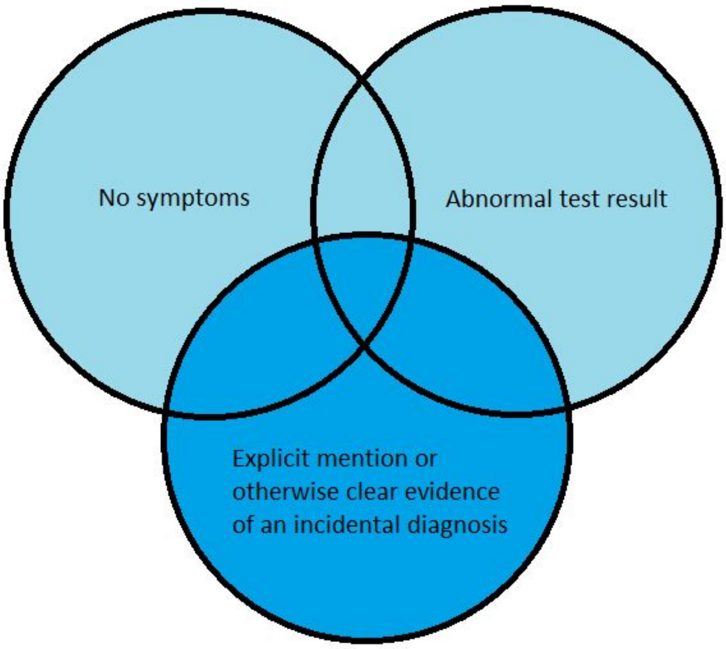

Figure 1 Visualisation of characteristics used to identify cases of incidentally diagnosed cancer. Areas shaded dark blue represent cases included in the main analysis, while areas in light blue indicate additional cases included in the sensitivity analysis.

Ethical approval

Ethical approval was not required given the anonymous nature of these data.

For peer review only - http://bmjopen.bmj.com/site/about/guidelines.xhtml

RESULTS

Incidentally diagnosed cancer patients

A total of 520/13,810 (4%) patients aged 15+ years were diagnosed incidentally with one of 25 cancer sites (other than prostate cancer). Men were more likely to be diagnosed incidentally than women (5% of men vs 3% of women), although there was no evidence to support this after adjustment for age and cancer site (see Table 1). The odds of being diagnosed incidentally with cancer generally increased with age (joint Wald test p-value = <0.001).

Crude and adjusted odds ratios indicated substantial variation in the odds of incidental diagnosis between cancer sites (see Figure 2 and Table 1). Almost a quarter (23%) of leukaemia patients and over a tenth (13%) of all renal cancer patients were diagnosed incidentally. More than a tenth of patients with thyroid (12%) and liver cancer (11%) were also diagnosed incidentally. In contrast, less than 1% of patients with endometrial, testicular, breast, sarcoma, brain, oesophageal and cervical cancers were diagnosed incidentally.

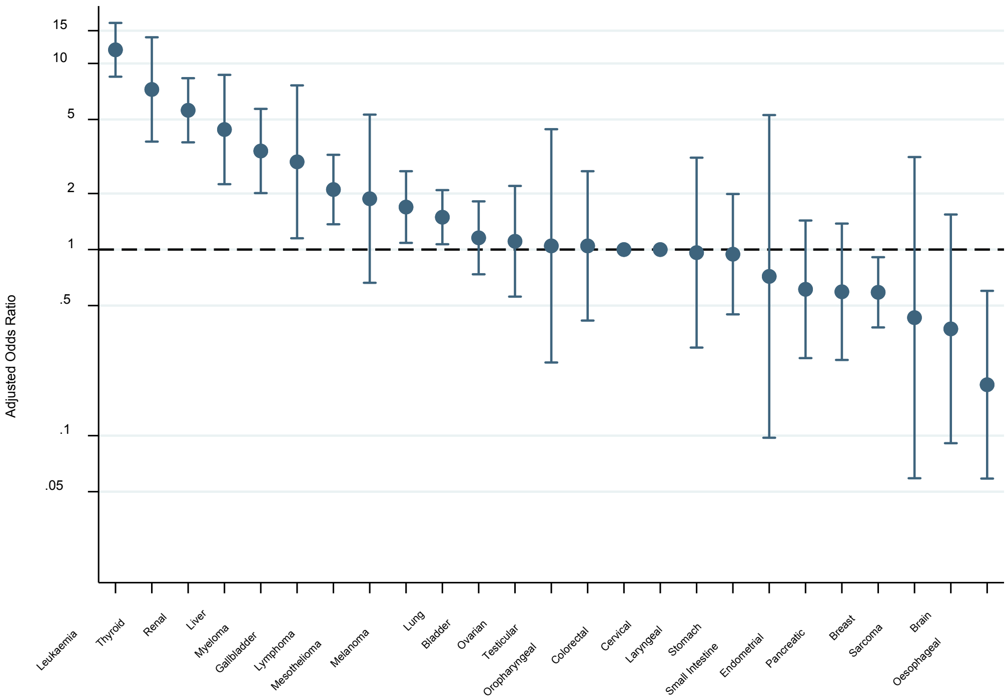

Figure 2 Odds ratios of incidental versus symptomatic diagnosis of cancer by cancer site (n=13,810; reference group: colorectal cancer). NB there is no odds ratio for ovarian cancer as there were no incidentally diagnosed cases of cervical cancer.

Table 1 Characteristics of incidental cancer patients versus non-incidental cancer patients, and crude/adjusted odds ratios of incidental status (n=13,810)

	Total	Incidental		Crude	Adjusted[a]
	N	n	% (95% CI)	OR (95% CI)	OR (95% CI)
Total	13810	520	4% (3–4%)	-	-
**Sex**				**0.001**[b]	0.204[b]
Men	5839	278	5% (4–5%)	Ref.	Ref.
Women	7971	242	3% (3–3%)	0.63 (0.53–0.75)	0.88 (0.72–1.07)
**Age group**				**<0.001**[b]	**<0.001**[b]
15–49 years	2072	31	1% (1–2%)	0.40 (0.27–0.59)	0.39 (0.26–0.60)
50–59 years	2050	65	3% (2–4%)	0.86 (0.63–1.17)	0.88 (0.64–1.21)
60–69 years	3181	117	4% (3–4%)	Ref.	Ref.
70–79 years	3656	170	5% (4–5%)	1.28 (1.00–1.62)	1.28 (1.00–1.64)
80+ years	2851	137	5% (4–6%)	1.32 (1.03–1.70)	1.45 (1.12–1.89)
**Cancer site**				**<0.001**[b]	**<0.001**[b]
Leukaemia	450	103	23% (19–27%)	10.49 (7.55–14.58)	11.84 (8.49–16.51)
Renal	356	46	13% (10–17%)	5.25 (3.53–7.78)	5.60 (3.77–8.33)
Thyroid	110	13	12% (7–19%)	4.74 (2.53–8.88)	7.25 (3.80–13.82)
Liver	103	11	11% (6–18%)	4.23 (2.16–8.27)	4.42 (2.24–8.68)
Myeloma	228	20	9% (6–13%)	3.40 (2.02–5.72)	3.39 (2.01–5.70)
Gallbladder	68	5	7% (3–16%)	2.81 (1.09–7.20)	2.96 (1.15–7.62)
Mesothelioma	75	4	5% (2–13%)	1.99 (0.71–5.61)	1.88 (0.66–5.31)
Lymphoma	698	33	5% (3–7%)	1.75 (1.14–2.69)	2.10 (1.37–3.23)
Vulval	73	3	4% (1–11%)	1.51 (0.46–4.94)	1.70 (0.52–5.60)
Lung	1875	77	4% (3–5%)	1.51 (1.08–2.12)	1.49 (1.07–2.09)
Melanoma	834	30	4% (3–5%)	1.32 (0.85–2.05)	1.69 (1.09–2.64)
Bladder	842	28	3% (2–5%)	1.22 (0.78–1.91)	1.16 (0.74–1.82)
Colorectal	2399	66	3% (2–3%)	Ref.	Ref.
Stomach	302	8	3% (1–5%)	0.96 (0.46–2.02)	0.94 (0.45–1.99)
Ovarian	394	10	3% (1–5%)	0.92 (0.47–1.81)	1.11 (0.56–2.20)
Laryngeal	121	3	2% (1–7%)	0.90 (0.28–2.90)	0.96 (0.30–3.12)
Oropharyngeal	213	5	2% (1–5%)	0.85 (0.34–2.13)	1.05 (0.42–2.64)
Small Intestine	53	1	2% (0.3–10%)	0.68 (0.09–4.99)	0.72 (0.10–5.28)
Pancreatic	370	6	2% (1–3%)	0.58 (0.25–1.35)	0.59 (0.26–1.38)
Endometrial	410	6	1% (1–3%)	0.52 (0.23–1.22)	0.61 (0.26–1.43)
Testicular	149	2	1% (0.4–5%)	0.48 (0.12–1.98)	1.05 (0.25–4.43)
Breast	2675	34	1% (1–2%)	0.46 (0.30–0.69)	0.59 (0.38–0.91)
Sarcoma	106	1	0.9% (0.2–5%)	0.34 (0.05–2.45)	0.43 (0.06–3.14)
Brain	215	2	0.9% (0.3–3%)	0.33 (0.08–1.36)	0.37 (0.09–1.54)
Oesophageal	566	3	0.5% (0.2–2%)	0.19 (0.06–0.60)	0.19 (0.06–0.60)
Cervical	125	0	0% (0–3%)	N/A	N/A

[a] adjusted for sex, age group, and cancer site
[b] joint Wald test p-value

Among the 520 incidentally diagnosed patients, a fifth (20%, 95% CI: 17–23%) were diagnosed with leukaemia, while other common cancer sites included lung (15%, 12–18%), colorectal (13%, 10–16%), and renal cancers (9%, 7–12%) (see Figure 3 and Table S2). There were 9 other cancer sites represented amongst the incidentally diagnosed cancer patient population with 10 or more patients each.

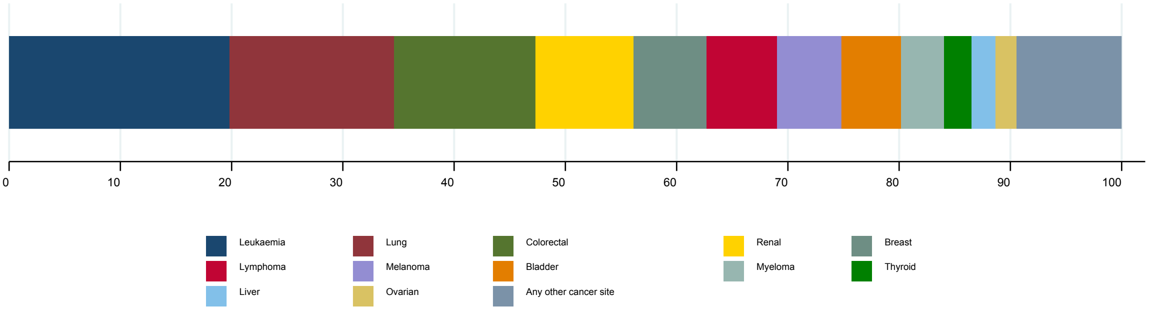

Figure 3 Commonly diagnosed cancer sites among the incidental cancer patient population; see Table S2 for frequencies

Sensitivity analyses (using a broader definition of incidental diagnosis) identified a further 272 cases, increasing the overall estimate of incidental diagnosis to 6% (see Table S3.1 and Figure S3.2). There was weak evidence to support greater odds of incidental diagnosis among men versus women (adjusted OR (95% CI): 0.84 (0.71–1.00)), with otherwise similar patterns of variation by age group and cancer site as in the main analysis.

For peer review only

Routes to incidental cancer diagnosis

We identified several clinical scenarios preceding an incidental diagnosis of cancer based on information available for 345 patients (66% of all incidentally diagnosed patients). These are outlined in Table 2 and discussed in further detail below.

Many patients received an incidental cancer diagnosis as a result of a clinical encounter for a pre-existing chronic disease in primary or secondary care. This included routine blood or urine testing, as part of chronic disease (or related risk factor) management and monitoring, which revealed abnormalities that led to the diagnosis of unsuspected cancer. Some patients were diagnosed following blood or imaging investigations before/after elective surgery for unrelated indications with a small number of patients where tumours were identified during surgery. A small number of patients were diagnosed after blood or imaging investigations conducted as part of follow up for a pre-existing cancer (e.g. scans to ascertain stage at diagnosis of prostate cancer led to the diagnosis of a renal cancer).

Other cancer patients were diagnosed following the investigation of unrelated acute conditions or presenting symptom(s) unlikely to be related to the subsequent cancer diagnosis. Several of these cases were being investigated for another suspected cancer (e.g. a CT scan for a suspected pelvic cancer leading to the diagnosis of colorectal cancer) but in others the diagnosis was more serendipitous (e.g. breast lump found on examination for chest infection).

Table 2 Clinical scenarios preceding the incidental diagnosis of cancer

Clinical scenario	Description and examples
Monitoring or managing pre-existing chronic morbidity	Blood or imaging investigations as part of monitoring or management of a chronic morbidity<br>E.g. haematuria on dipstick urine testing led to diagnosis of bladder cancer<br>E.g. annual blood tests for hypertension led to diagnosis of leukaemia
Before/after surgery	Blood or imaging investigations conducted before or after surgery, or more rarely, tumours identified during elective surgery for unrelated condition<br>E.g. pre-operative chest x-ray leading to diagnosis of lung cancer<br>E.g. microscopic haematuria pre-cataract operation leading to diagnosis of a urological cancer
Follow up of a pre-existing cancer	Blood or imaging investigations conducted as part of follow up for a pre-existing cancer<br>E.g. scans to ascertain stage at diagnosis of prostate cancer leading to the diagnosis of a urological cancer
Investigation of unrelated acute or new condition or symptoms	Blood or imaging investigations for a new symptom or otherwise acute condition<br>E.g. an abdominal ultrasound scan for dyspepsia leading to the diagnosis of a urological cancer<br>E.g. abnormal result or irregular mole noted during health check

For peer review only

DISCUSSION

Principal findings

Around 1 in 25 cancer patients in our study population were diagnosed incidentally, with a preponderance among older patients, and patients with leukaemia, renal cancer, thyroid cancer, liver cancer, and multiple myeloma. Several clinical scenarios that preceded incidental diagnosis include healthcare encounters due to previously known conditions, and the investigation of acute or new conditions unrelated to cancer.

Strengths and limitations

Information on incidental status at diagnosis is not routinely recorded as part of cancer registration, healthcare records, or other administrative databases. A strength of our study is that it provides unique evidence about this less well documented diagnostic pathway of cancer, among a large and representative incident cohort of cancer patients. Furthermore, we have identified incidentally diagnosed patients using a novel approach, based on the triangulation of information regarding symptom status, test results, and explicit mention of incidental diagnosis (Figure 1).

Nevertheless, interpretation of the findings should be mindful of the secondary nature of our analysis. Our definition of incidentally diagnosed cancer was deliberately conservative, designed to maximise specificity and reduce the likelihood of patients being mistakenly identified as incidental diagnoses. However, this may have led to the under-estimation of cases, which motivated our sensitivity analysis.

Comparison with existing literature

Literature examining incidentally diagnosed cancer is limited, although some evidence may be gleaned from studies on incidental findings detected in the context of research studies. Estimates of clinically important incidental findings (including cancer but also other diseases) vary substantially depending on imaging field (whole body, or specific organ) and modality however, and participants of research studies are unlikely to be representative of the general population [19,20].

Though we were unable to examine potential overdiagnosis, we identified notable proportions of incidentally diagnosed patients with thyroid and renal cancer, and melanoma patients. This is consistent with prior evidence indicating potential over-diagnosis of these cancers [21–24]. A few studies have examined clinical scenarios that result in incidental diagnosis of individual cancer sites such as melanoma, lung cancer, and renal cancer [17,25–27]. A study examining self-reported symptoms of haematological cancer patients found that a third of patients did not report any symptoms before diagnosis, with chronic lymphocytic leukaemia patients being particularly prone to being diagnosed incidentally, for example through blood tests at routine healthcare encounters [28]. Our findings are consistent with the findings of these studies, but additionally suggest that incidental diagnosis occurs across a range of common and rarer cancers.

Implications

Our findings indicate that a substantial proportion of cancer patients are diagnosed with cancer incidentally, without having presented with symptoms related to the subsequent diagnosis. An incidental cancer diagnosis could represent fortuitous early diagnosis of an invasive tumour, and therefore be of clinical benefit for a proportion of patients. However it could also represent overdiagnosis, which could lead to considerable psychological morbidity and unnecessary treatment.

We identified several clinical scenarios that resulted in the incidental diagnosis of cancer; their frequency is likely to be affected by system level factors such as approaches to chronic disease

For peer review only

monitoring, incentives and thresholds for investigation, availability of imaging services, and rates of elective surgery [29,30]. Given increasing levels of multi-morbidity and an ageing population, there is progressively greater use of blood-based testing and imaging studies, which could lead to a greater proportion of cancer patients being diagnosed incidentally [10]. Relatedly, incidental diagnosis of cancer occurred during investigation or follow up of a pre-existing (unrelated) tumour in a small number of patients. As the survival of patients with cancer continues to improve, this could also become a more prevalent route to incidental diagnosis [31].

Conclusions

In conclusion, we have provided evidence about the frequency and common scenarios leading to incidental diagnosis of cancer. Our findings indicate that this is likely to affect around one in 25 cancer patients. Establishing the prognostic, psychosocial and economic implications of incidental diagnosis of cancer is necessary, given the increasing availability of preventive healthcare services for chronic diseases, and ageing populations.

For peer review only

References

1 Elliss-Brookes L, McPhail S, Ives A, *et al.* Routes to diagnosis for cancer - determining the patient journey using multiple routine data sets. *Br J Cancer* 2012;**107**:1220–6. doi:10.1038/bjc.2012.408

2 Jensen H, Tørring ML, Olesen F, *et al.* Cancer suspicion in general practice, urgent referral and time to diagnosis: a population-based GP survey and registry study. *BMC Cancer* 2014;**14**:636. doi:10.1186/1471-2407-14-636

3 Lumbreras B, Donat L, Hernández-Aguado I. Incidental findings in imaging diagnostic tests: a systematic review. *Br J Radiol* 2010;**83**:276–89. doi:10.1259/bjr/98067945

4 Kroczek EK, Wieners G, Steffen I, *et al.* Non-traumatic incidental findings in patients undergoing whole-body computed tomography at initial emergency admission. *Emerg Med J* 2017;:emermed-2016-205722. doi:10.1136/emermed-2016-205722

5 O'Sullivan JW, Muntinga T, Grigg S, *et al.* Prevalence and outcomes of incidental imaging findings: umbrella review. *BMJ* 2018;:k2387. doi:10.1136/bmj.k2387

6 Maskell G. Think before you scan. *Bmj* 2018;**3754**:k3754. doi:10.1136/bmj.k3754

7 NHS. NHS Health Check. 2017.http://www.healthcheck.nhs.uk/

8 NHS Digital. Quality and Outcomes Framework. Prim. Care. 2016.http://content.digital.nhs.uk/QOF

9 Treadwell J, McCartney M. Overdiagnosis and overtreatment: generalists--it's time for a grassroots revolution. *Br J Gen Pract* 2016;**66**:116–7. doi:10.3399/bjgp16X683881

10 O'Sullivan JW, Stevens S, Hobbs FDR, *et al.* Temporal trends in use of tests in UK primary care, 2000-15: retrospective analysis of 250 million tests. *Bmj* 2018;:k4666. doi:10.1136/bmj.k4666

11 Esserman LJ, Thompson IM, Reid B, *et al.* Addressing overdiagnosis and overtreatment in cancer: A prescription for change. *Lancet Oncol* 2014;**15**:e234–42. doi:10.1016/S1470-2045(13)70598-9

12 Jenniskens K, de Groot JAH, Reitsma JB, *et al.* Overdiagnosis across medical disciplines: a scoping review. *BMJ Open* 2017;**7**:e018448. doi:10.1136/bmjopen-2017-018448

13 Davies L, Petitti DB, Martin L, *et al.* Defining, Estimating, and Communicating Overdiagnosis in Cancer Screening. *Ann Intern Med* 2018;**169**:36. doi:10.7326/M18-0694

14 Rubin GP, McPhail S, Elliot K, *et al.* National Audit of Cancer Diagnosis in Primary Care. London: 2011. http://www.rcgp.org.uk/policy/rcgp-policy-areas/national-audit-of-cancer-diagnosis-in-primary-care.aspx

15 Lyratzopoulos G, Abel GA, McPhail S, *et al.* Gender inequalities in the promptness of diagnosis of bladder and renal cancer after symptomatic presentation: evidence from secondary analysis of an English primary care audit survey. *BMJ Open* 2013;**3**:e002861. doi:10.1136/bmjopen-2013-002861

16 Davies L, Ouellette M, Hunter M, *et al.* The increasing incidence of small thyroid cancers: Where are the cases coming from? *Laryngoscope* 2010;**120**:2446–51. doi:10.1002/lary.21076

17 Kocher F, Lunger F, Seeber A, *et al.* Incidental Diagnosis of Asymptomatic Non-Small-Cell Lung Cancer: A Registry-Based Analysis. *Clin Lung Cancer* 2016;**17**:62–7. doi:10.1016/j.cllc.2015.08.006

For peer review only

18 NICE. Prostate cancer : diagnosis and management (CG175). Published Online First: 2014.https://www.nice.org.uk/guidance/cg175

19 The Royal College of Radiologists. Management of Incidental Findings Detected During Research Imaging. 2011. papers3://publication/uuid/627D4627-FE73-472B-BD26-9FD0F38FC547

20 Booth TC, Jackson A, Wardlaw JM, *et al.* Incidental findings found in 'healthy' volunteers during imaging performed for research: Current legal and ethical implications. *Br J Radiol* 2010;**83**:456–65. doi:10.1259/bjr/15877332

21 Weyers W. The 'epidemic' of melanoma between under- and overdiagnosis. *J Cutan Pathol* 2012;**39**:9–16. doi:10.1111/j.1600-0560.2011.01831.x

22 Welch HG, Black WC. Overdiagnosis in cancer. *J Natl Cancer Inst* 2010;**102**:605–13. doi:10.1093/jnci/djq099

23 Moynihan R, Doust J, Henry D. Preventing overdiagnosis: how to stop harming the healthy. *Bmj* 2012;**344**:e3502–e3502. doi:10.1136/bmj.e3502

24 Ahn HS, Kim HJ, Welch HG. Korea's Thyroid-Cancer "Epidemic" — Screening and Overdiagnosis. *N Engl J Med* 2014;**371**:1765–7. doi:10.1056/NEJMp1409841

25 Cufari ME, Proli C, Phull M, *et al.* Increasing incidence of non-smoking lung cancer: presentation of patients with early disease to a tertiary institution in the UK. *Lung Cancer* 2016;**91**:S17–8. doi:10.1016/S0169-5002(16)30066-6

26 Avilés-Izquierdo JA, Molina-López I, Rodríguez-Lomba E, *et al.* Who detects melanoma? Impact of detection patterns on characteristics and prognosis of patients with melanoma. *J Am Acad Dermatol* 2016;:1–8. doi:10.1016/j.jaad.2016.07.009

27 Hofbauer SL, de Martino M, Seemann C, *et al.* Associations between presenting symptoms, clinicopathological parameters, and prognosis in a contemporary series of patients with renal cell carcinoma. *Korean J Urol* 2014;**55**:505–10. doi:10.4111/kju.2014.55.8.505

28 Howell DA, Smith AG, Jack A, *et al.* Time-to-diagnosis and symptoms of myeloma, lymphomas and leukaemias: a report from the Haematological Malignancy Research Network. *BMC Blood Disord* 2013;**13**:9. doi:10.1186/2052-1839-13-9

29 Pollack CE, Soulos PR, Herrin J, *et al.* The Impact of Social Contagion on Physician Adoption of Advanced Imaging Tests in Breast Cancer. *J Natl Cancer Inst* 2017;**109**:1–8. doi:10.1093/jnci/djw330

30 Barraclough K. New NICE guidance on referral for cancer. *BMJ* 2015;**351**:h3640. doi:10.1136/bmj.h3640

31 Murphy CC, Gerber DE, Pruitt SL. Prevalence of Prior Cancer Among Persons Newly Diagnosed With Cancer. *JAMA Oncol* 2017;**75390**:1–4. doi:10.1001/jamaoncol.2017.3605

For peer review only

Additional information

Acknowledgements

We are grateful to all general practitioners and healthcare professionals that were involved in the collection and submission of anonymous data to the audit, and to the respective Cancer Networks, the Royal College of General Practitioners, the former National Cancer Action Team and the former National Clinical Intelligence Network (NCIN) of Public Health England (PHE) for supporting the audit.

Competing interests

The authors have declared no competing interests.

Funding statement

This work was supported by the UK Department of Health as part of the programme of the Policy Research Unit in Cancer Awareness, Screening and Early Diagnosis [grant number no. 106/0001]. The Policy Research Unit in Cancer Awareness, Screening, and Early Diagnosis is a collaboration between researchers from seven institutions (Queen Mary University of London, University College London, King's College London, London School of Hygiene and Tropical Medicine, Hull York Medical School, Durham University and Peninsula Medical School/University of Exeter). GL is supported by Cancer Research UK Clinician Advanced Scientist Fellowship [grant number: C18081/A18180]. The views expressed are those of the authors and not necessarily those of the Department of Health or Cancer Research UK. The funders of the study had no role in the study design, data analysis, data interpretation, or writing of the report. The corresponding author had full access to all the data in the study and had final responsibility for the decision to submit for publication.

Authorship contribution

MMK, GPR, and GL conceived the study. MMK conducted all statistical analyses with assistance from GL. MMK wrote the first draft of the manuscript, and prepared the tables and figures, supervised by GL. MMK, GPR, SMcP, and GL contributed to the interpretation of the results, revised the manuscript and approved the final version of the manuscript.

For peer review only

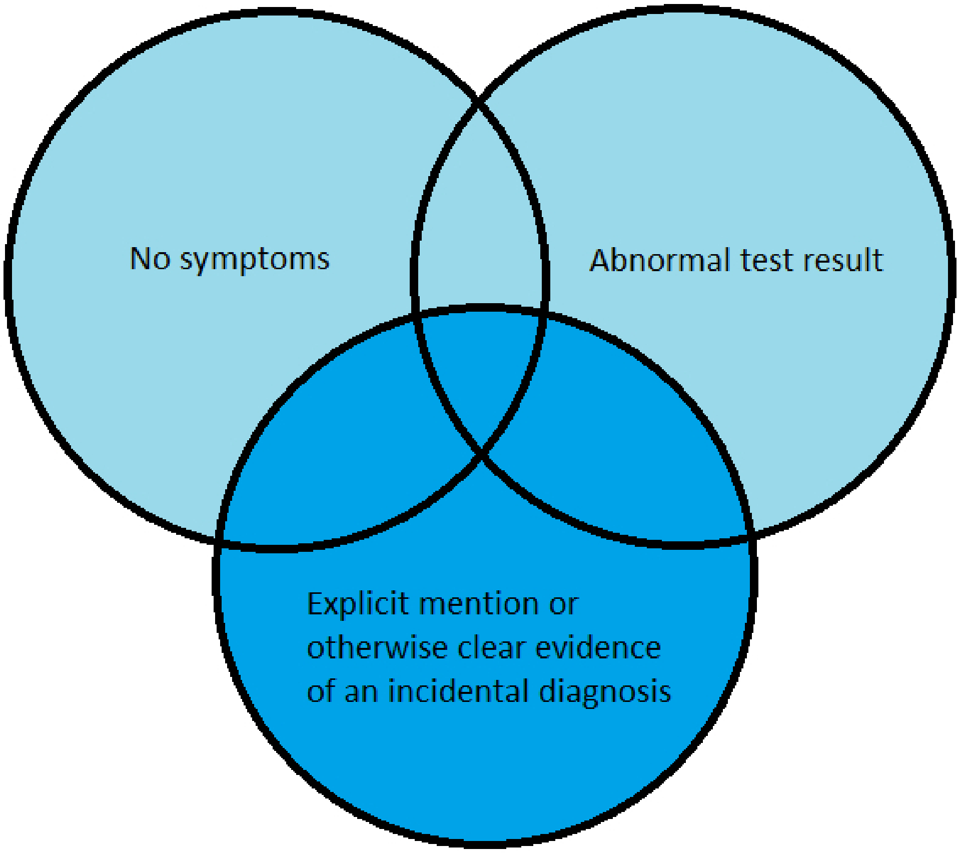

Figure 1: Visualisation of characteristics used to identify cases of incidentally diagnosed cancer. Areas shaded dark blue represent cases included in the main analysis, while areas in light blue indicate additional cases included in the sensitivity analysis.

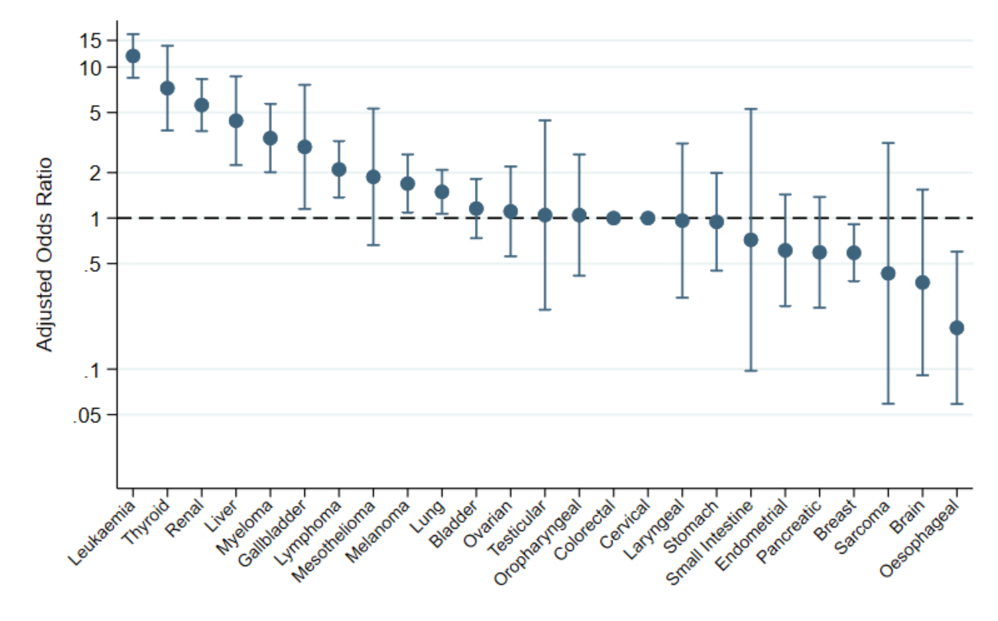

Figure 2: Odds ratios of incidental versus symptomatic diagnosis of cancer by cancer site (n=13,810; reference group: colorectal cancer). NB there is no odds ratio for ovarian cancer as there were no incidentally diagnosed cases of cervical cancer.

282x176mm (72 x 72 DPI)

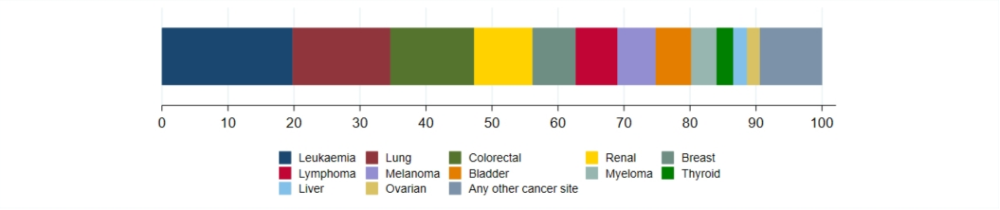

Figure 3: Commonly diagnosed cancer sites among the incidental cancer patient population; see Table S2 for frequencies

423x88mm (72 x 72 DPI)

Supplementary information for "*Incidentally diagnosed cancer: population-based evidence on frequency, variation, and commonly preceding clinical scenarios*" Koo et al., 2018

Table of contents

- Figure S1: Flow chart describing sample derivation for main analysis
- Table S2: Cancer site case-mix of incidentally diagnosed cancer patients
- S3: Sensitivity analysis using a broader definition of incidental diagnosis
  - Table S3.1 Characteristics of incidental cancer patients versus non-incidental cancer patients, and crude/adjusted odds ratios of incidental status (n=14,082)
  - Figure S3.2 Odds ratios of incidental versus symptomatic diagnosis of cancer by cancer site (n=14,082; reference group: colorectal cancer)

For peer review only

Supplementary files for Koo et al., 2018

Figure S1: Flow chart describing sample derivation for main analysis

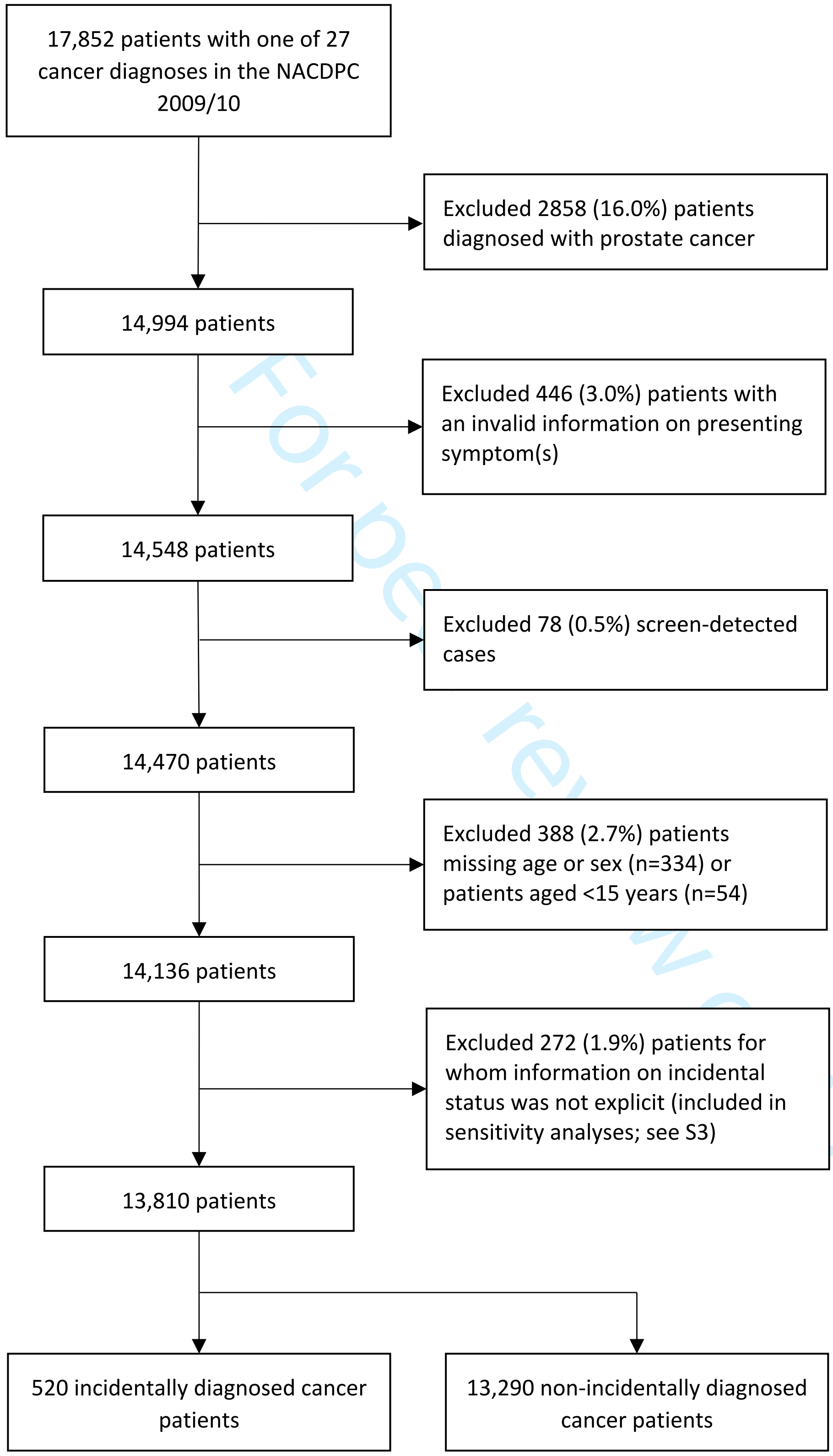

Supplementary files for Koo et al., 2018

Table S2: Cancer site case-mix of incidentally diagnosed cancer patients

Cancer	N	% (95% CI)
Leukaemia	103	20% (17–23%)
Lung	77	15% (12–18%)
Colorectal	66	13% (10–16%)
Renal	46	9% (7–12%)
Breast	34	7% (5–9%)
Lymphoma	33	6% (5–9%)
Melanoma	30	6% (4–8%)
Bladder	28	5% (4–8%)
Myeloma	20	4% (3–6%)
Thyroid	13	3% (1–4%)
Liver	11	2.1% (1.2–3.7%)
Ovarian	10	1.9% (1.0–3.5%)
Stomach	8	1.5% (0.8–3.0%)
Endometrial	6	1.2% (0.5–2.5%)
Pancreatic	6	1.2% (0.5–2.5%)
Gallbladder	5	1.0% (0.4–2.2%)
Oropharyngeal	5	1.0% (0.4–2.2%)
Mesothelioma	4	0.8% (0.3–2.0%)
Laryngeal	3	0.6% (0.2–1.7%)
Oesophageal	3	0.6% (0.2–1.7%)
Vulval	3	0.6% (0.2–1.7%)
Brain	2	0.4% (0.1–1.4%)
Testicular	2	0.4% (0.1–1.4%)
Sarcoma	1	0.2% (0.03–1.1%)
Small Intestine	1	0.2% (0.03–1.1%)
Total	520	100%

* Proportion of patients with each cancer site, of the total incidentally diagnosed population (n=520)

For peer review only

Supplementary files for Koo et al., 2018

S3: Sensitivity analysis with broader definition of incidental diagnosis

Table S3.1 Characteristics of incidental cancer patients versus non-incidental cancer patients, and crude/adjusted odds ratios of incidental status (n=14,082)

	Total	Incidental		Crude	Adjusted[a]
	N	n	% (95% CI)	OR (95% CI)	OR (95% CI)
Total	14082	792	6% (5–6%)	-	-
**Sex**				**0.001[b]**	**0.045[b]**
Men	5983	422	7% (6–8%)	Ref.	Ref.
Women	8099	370	5% (4–5%)	0.63 (0.55–0.73)	0.84 (0.71–1.00)
**Age group**				**<0.001[b]**	**<0.001[b]**
15–49 years	2089	48	2% (2–3%)	0.36 (0.26–0.50)	0.36 (0.26–0.51)
50–59 years	2080	95	5% (4–6%)	0.73 (0.57–0.94)	0.75 (0.58–0.98)
60–69 years	3264	200	6% (5–7%)	Ref.	Ref.
70–79 years	3739	253	7% (6–8%)	1.11 (0.92–1.35)	1.08 (0.89–1.33)
80+ years	2910	196	7% (6–8%)	1.11 (0.90–1.36)	1.17 (0.94–1.45)
**Cancer site**				**<0.001[b]**	**<0.001[b]**
Leukaemia	511	164	32% (28–36%)	11.25 (8.55–14.81)	12.48 (9.46–16.48)
Liver	116	24	21% (14–29%)	6.21 (3.79–10.16)	6.28 (3.82–10.32)
Renal	373	63	17% (13–21%)	4.84 (3.45–6.78)	5.12 (3.64–7.19)
Thyroid	113	16	14% (9–22%)	3.93 (2.23–6.92)	5.93 (3.31–10.60)
Myeloma	241	33	14% (10–19%)	3.78 (2.48–5.74)	3.74 (2.46–5.69)
Gallbladder	68	5	7% (3–16%)	1.89 (0.74–4.80)	2.00 (0.78–5.09)
Bladder	869	55	6% (5–8%)	1.61 (1.15–2.26)	1.52 (1.08–2.14)
Lung	1913	115	6% (5–7%)	1.52 (1.15–2.01)	1.50 (1.14–1.97)
Lymphoma	704	39	6% (4–7%)	1.40 (0.95–2.04)	1.64 (1.12–2.41)
Mesothelioma	75	4	5% (2–13%)	1.34 (0.48–3.75)	1.23 (0.44–3.45)
Melanoma	839	35	4% (3–6%)	1.04 (0.70–1.54)	1.30 (0.88–1.94)
Vulval	73	3	4% (1–11%)	1.02 (0.32–3.30)	1.19 (0.37–3.87)
Colorectal	2431	98	4% (3–5%)	Ref.	Ref.
Ovarian	398	14	4% (2–6%)	0.87 (0.49–1.54)	1.05 (0.59–1.88)
Stomach	303	9	3% (2–6%)	0.73 (0.36–1.46)	0.72 (0.36–1.44)
Breast	2717	76	3% (2–3%)	0.69 (0.51–0.93)	0.91 (0.66–1.25)
Pancreatic	374	10	3% (1–5%)	0.65 (0.34–1.27)	0.66 (0.34–1.28)
Laryngeal	121	3	2% (1–7%)	0.61 (0.19–1.94)	0.62 (0.19–1.98)
Oropharyngeal	213	5	2% (1–5%)	0.57 (0.23–1.42)	0.68 (0.27–1.69)
Endometrial	413	9	2% (1–4%)	0.53 (0.27–1.06)	0.62 (0.31–1.24)
Small Intestine	53	1	2% (–10%)	0.46 (0.06–3.35)	0.48 (0.07–3.55)
Sarcoma	107	2	2% (1–7%)	0.45 (0.11–1.86)	0.57 (0.14–2.36)
Testicular	149	2	1% (–5%)	0.32 (0.08–1.33)	0.66 (0.16–2.76)
Brain	215	2	1% (–3%)	0.22 (0.05–0.91)	0.25 (0.06–1.02)
Cervical	126	1	1% (–4%)	0.19 (0.03–1.38)	0.29 (0.04–2.09)
Oesophageal	567	4	1% (–2%)	0.17 (0.06–0.46)	0.17 (0.06–0.45)

[a] adjusted for sex, age group, and cancer site
[b] joint Wald test p-value

For peer review only

Supplementary files for Koo et al., 2018

Figure S3.2 Odds ratios of incidental versus symptomatic diagnosis of cancer by cancer site (n=14,082; reference group: colorectal cancer)

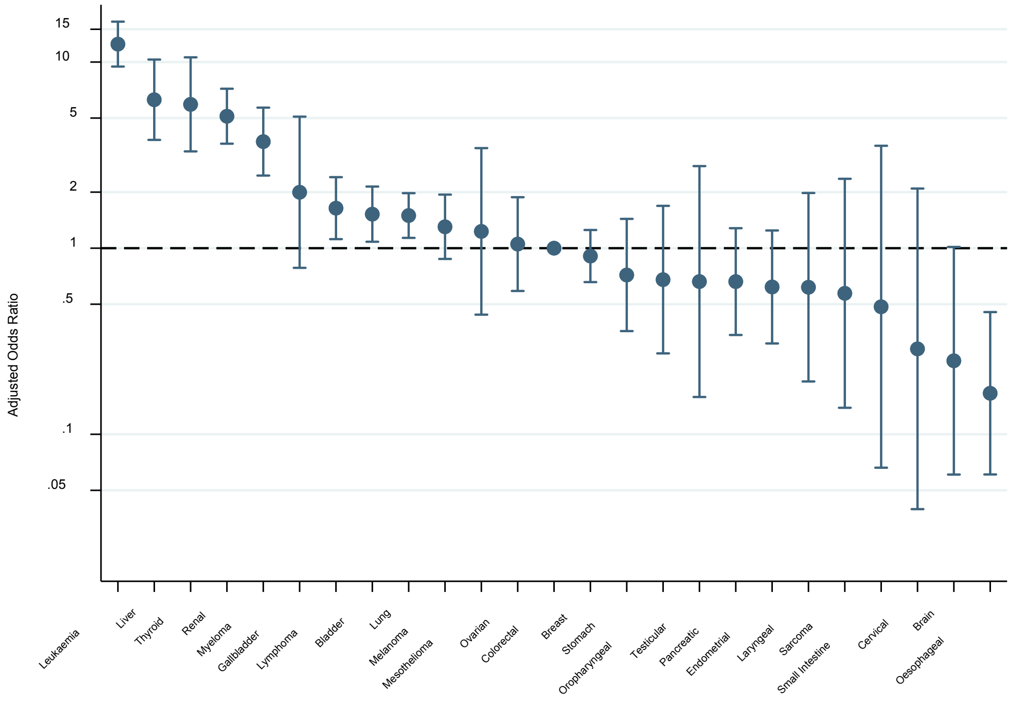

BMJ Open

Incidentally diagnosed cancer and commonly preceding clinical scenarios: a cross-sectional descriptive analysis of English audit data

Journal:	*BMJ Open*
Manuscript ID	bmjopen-2018-028362.R1
Article Type:	Research
Date Submitted by the Author:	26-Mar-2019
Complete List of Authors:	Koo, Minjoung; UCL, Behavioural Science and Health<br>Rubin, Greg; Royal Victoria Infirmary, Institute of Health and Society, Newcastle University<br>McPhail, Sean; Public Health England, NCRAS; UCL, Behavioural Science and Health<br>Lyratzopoulos, Georgios ; UCL, Behavioural Science and Health; Public Health England, NCRAS
**Primary Subject Heading**:	Health services research
Secondary Subject Heading:	Radiology and imaging, Oncology, Diagnostics, General practice / Family practice, Haematology (incl blood transfusion)
Keywords:	PRIMARY CARE, RADIOLOGY & IMAGING, PUBLIC HEALTH, HEALTH SERVICES ADMINISTRATION & MANAGEMENT, ONCOLOGY, PATHOLOGY

SCHOLARONE™
Manuscripts

Incidentally diagnosed cancer and commonly preceding clinical scenarios: a cross-sectional descriptive analysis of English audit data

Minjoung Monica Koo PhD [a], Greg P Rubin MD [b], Sean McPhail PhD [a,c], & Georgios Lyratzopoulos MD [a,c]

Affiliations:
[a] University College London, Gower street, London WC1E 6BT, UK
[b] Institute of Health and Society, Newcastle University, Sir James Spence Institute, Royal Victoria Infirmary, Newcastle upon Tyne, NE1 4LP, UK
[c] National Cancer Registration and Analysis Service, Public Health England, Wellington House, 133-155 Waterloo Road, London, SE1 8UG, UK

Corresponding Author:
Dr Minjoung Monica Koo (monica.koo.14@ucl.ac.uk)

Funding statement:
This work was supported by a grant from the UK Department of Health [grant number no. 106/0001], as part of the program of the Policy Research Unit in Cancer Awareness, Screening and Early Diagnosis. The Policy Research Unit in Cancer Awareness, Screening, and Early Diagnosis receives funding for a research program from the Department of Health Policy Research Programme. It is a collaboration between researchers from seven institutions (Queen Mary University of London, University College London, King's College London, London School of Hygiene and Tropical Medicine, Hull York Medical School, Durham University, and Peninsula Medical School/University of Exeter). GL is supported by a Cancer Research UK Advanced Clinician Scientist Fellowship [grant number: C18081/A18180]. The views expressed are those of the authors and not necessarily those of the Department of Health or Cancer Research UK.

Competing interests:
None declared

Abstract: 270
Main text: 2150
References: 33
Tables: 2
Figures: 3

For peer review only

ABSTRACT

Objectives

Cancer can be diagnosed in the absence of tumour-related symptoms, but little is known about the frequency and circumstances preceding such diagnoses outside participation in screening programmes. We aimed to examine incidentally diagnosed cancer among a cohort of cancer patients diagnosed in England.

Design

Cross-sectional study of national primary care audit data on incident cancer patients.

Setting

We analysed free-text information on cancer patients aged 15 or older included in the English National Audit of Cancer Diagnosis in Primary Care (NACDPC) (2009-10). Patients with screen-detected cancers and those diagnosed with prostate cancer were excluded. We examined the odds of incidental cancer diagnosis by patient characteristics and cancer site using logistic regression, and described clinical scenarios leading to incidental diagnosis.

Results

Among the studied cancer patient population (n=13,810), 520 (4%) patients were diagnosed incidentally. The odds of incidental cancer diagnosis increased with age ($p<0.001$), without a difference between men and women after adjustment. Incidental diagnosis was most common among patients with leukaemia (23%), renal (13%) and thyroid cancer (12%), and least common among patients with brain (0.9%), oesophageal (0.5%), and cervical cancer (no cases). Variation in odds of incidental diagnosis by cancer site remained after adjusting for age group and sex.

Incidental diagnoses were commonly preceded by a range of clinical scenarios across primary and secondary care. These included the monitoring or management of pre-existing conditions, routine testing before or after elective surgery, and the investigation of unrelated acute or new conditions.

Conclusions

One in 25 patients with cancer in our population-based cohort were diagnosed incidentally. The epidemiological, clinical, psychological, and economic implications of this diagnostic route merit further investigation.

For peer review only

Strengths and limitations of this study

- The findings are based on a large population-based cohort of individuals diagnosed with a range of cancers
- Diagnostic status (incidental or non-incidental) was identified using free-text information provided by primary care physicians based on primary care records
- We describe common mechanisms of incidental diagnosis beyond a single modality or cancer site with a high level of detail
- We were unable to examine differences in clinical outcome between incidental and non-incidentally diagnosed cancer patients

For peer review only

INTRODUCTION

Cancer is most often diagnosed following presentation with symptoms caused by the malignancy [1,2]. However, some patients are diagnosed with cancer incidentally, in the absence of symptoms that could not plausibly be related to the tumour and outside formal cancer screening or surveillance activity. The use of imaging technologies (including x-ray, CT, MRI, and PET scans) is one of the commonly described routes to incidental diagnosis of different diseases, including cancer [3–6]. Chronic disease management involving periodic routine blood or urine testing may represent another common pathway to incidental diagnosis and are increasingly used in primary care [7–10]. Nonetheless, evidence regarding the frequency of such incidental diagnoses is currently limited.

Since incidental diagnoses are characterised by the absence of tumour related symptoms, it is plausible that some patients with incidentally detected cancer could be overdiagnosed, whereby the detected cancer would not have otherwise caused symptoms in the patient's lifetime [11]. Concerns about overdiagnosis thus far have largely focused on screening-detected cancers (e.g. breast cancer), but it may be also occurring in other contexts [12,13]. Ahead of considering the clinical, psychological, or economic consequences associated with incidental diagnosis (including the potential for overdiagnosis), we need to address gaps in our knowledge about the frequency and characteristics of incidentally diagnosed cancer.

We therefore aimed to examine the frequency of incidental diagnosis among an incident cohort of cancer patients; compare the characteristics of incidentally vs non-incidentally diagnosed patients; and examine common pathways and mechanisms likely to lead to incidental diagnosis of cancer.

For peer review only

METHODS

Study design and population

We analysed cross-sectional data collected as part of the English National Audit of Cancer Diagnosis in Primary Care (NACDPC) [14]. Briefly, health professionals from 1,170 participating general practices (representing 14% of practices in England) provided information on the diagnostic pathway for a consecutive sample of patients diagnosed with cancer during April 2009–2010. Participating practices were comparable to non-participating practices in (former) respective Cancer Networks, and the patient population was broadly representative of the contemporary national incident cancer patient cohort [14,15]. Clinicians participating in the NACDPC provided information regarding the main presenting symptoms, cancer diagnosis, demographic characteristics, and route of diagnosis based on primary care records.

Definition and identification of cases

The nature of cancer diagnosis (incidental or non-incidental), was ascertained by examination of the free-text information included in the presenting symptoms data field (answering the audit question "what were the main presenting symptom(s) [of the patient]?").

Informed by previous literature, we defined the incidental diagnosis of cancer as the diagnosis of cancer in individuals declared as asymptomatic outside the context of population-based screening participation by NACDPC auditors, or individuals noted to have symptoms or clinical signs at presentation that had not been the initial reason for encounter [5,16,17]. Cases were initially identified by MMK, and subsequently reviewed and validated by GL and GPR; disagreements were resolved by discussion. Additionally, we identified 520 cases where there was an explicit mention of the incidental nature of diagnosis (e.g. by use of phrases including "accidental finding"; "chance finding"; "incidental"; "opportunistic" or other details regarding circumstances indicating an incidental diagnosis).

Patients diagnosed with prostate cancer were excluded *a priori*, given the difficulties in reliably distinguishing reasons for Prostate Specific Antigen testing [18]. Patients with screen-detected breast, colorectal, and cervical cancer, and those diagnosed through surveillance for pre-malignant or high-risk conditions were also excluded. Therefore, the study population comprised 13,810 patients aged 15 or older with sufficient information to determine incidental/non-incidental status, and complete information on cancer diagnosis, age group, and sex (see Supplementary Figure 1 for sample derivation).

Data analysis

Firstly, we compared the demographic and clinical characteristics of incidentally and non-incidentally diagnosed patients. Logistic regression was used to calculate crude and adjusted odds ratios of incidental diagnosis by sex, age group, and cancer site. We also examined the cancer site case-mix ('cancer site signature') of the incidentally diagnosed population, i.e. the relatively frequency of each cancer site among incidentally diagnosed patients. Colorectal cancer was used as the reference category for cancer site, as the most common non-gender specific cancer in our population. All statistical analyses were conducted in STATA SE v.15 (StataCorp, College Station, TX, USA).

Subsequently, we identified common clinical scenarios leading to incidental diagnosis based on a subgroup of patients with relevant information (n=345, 66% of all incidental diagnoses). These findings were synthesised narratively.

For peer review only

Sensitivity analysis

The definition of incidentally diagnosed cancer used in the main analysis was based on explicit reference to the incidental nature of cancer diagnosis (see dark blue shaded area in Figure 1). We performed sensitivity analyses expanding the definition of incidental cancer to include an additional 272 patients who were described as having had no symptoms (not otherwise specified), or an abnormal clinical findings (not otherwise specified), or both no symptoms and abnormal clinical findings to the same audit question “what were the main presenting symptom [of the patient]?” (indicated by the light blue shaded area in Figure 1).

Ethical approval

Ethical approval was not required given the anonymous nature of these data.

Patient and public involvement

Patients and members of the public were not involved in the design of this study.

For peer review only

RESULTS

Incidentally diagnosed cancer patients

A total of 520/13,810 (4%) patients aged 15+ years were diagnosed incidentally with one of 25 cancer sites (other than prostate cancer). Men were more likely to be diagnosed incidentally than women (5% of men vs 3% of women), although there was no evidence to support this after adjustment for age and cancer site (see Table 1). The odds of being diagnosed incidentally with cancer generally increased with age (joint Wald test p-value = <0.001).

Crude and adjusted odds ratios indicated substantial variation in the odds of incidental diagnosis between cancer sites (see Figure 2 and Table 1). Almost a quarter (23%) of leukaemia patients and over a tenth (13%) of all renal cancer patients were diagnosed incidentally. More than a tenth of patients with thyroid (12%) and liver cancer (11%) were also diagnosed incidentally. In contrast, less than 1% of patients with endometrial, testicular, breast, sarcoma, brain, oesophageal and cervical cancers were diagnosed incidentally.

For peer review only

Table 1 Characteristics of incidental cancer patients versus non-incidental cancer patients, and crude/adjusted odds ratios of incidental status (n=13,810)

	Total	Incidental		Crude	Adjusted[a]
	N	n	% (95% CI)	OR (95% CI)	OR (95% CI)
Total	13810	520	4% (3–4%)	-	-
**Sex**				**0.001[b]**	0.204[b]
Men	5839	278	5% (4–5%)	Ref.	Ref.
Women	7971	242	3% (3–3%)	0.63 (0.53–0.75)	0.88 (0.72–1.07)
**Age group**				**<0.001[b]**	**<0.001[b]**
15–49 years	2072	31	1% (1–2%)	0.40 (0.27–0.59)	0.39 (0.26–0.60)
50–59 years	2050	65	3% (2–4%)	0.86 (0.63–1.17)	0.88 (0.64–1.21)
60–69 years	3181	117	4% (3–4%)	Ref.	Ref.
70–79 years	3656	170	5% (4–5%)	1.28 (1.00–1.62)	1.28 (1.00–1.64)
80+ years	2851	137	5% (4–6%)	1.32 (1.03–1.70)	1.45 (1.12–1.89)
**Cancer site**				**<0.001[b]**	**<0.001[b]**
Leukaemia	450	103	23% (19–27%)	10.49 (7.55–14.58)	11.84 (8.49–16.51)
Renal	356	46	13% (10–17%)	5.25 (3.53–7.78)	5.60 (3.77–8.33)
Thyroid	110	13	12% (7–19%)	4.74 (2.53–8.88)	7.25 (3.80–13.82)
Liver	103	11	11% (6–18%)	4.23 (2.16–8.27)	4.42 (2.24–8.68)
Myeloma	228	20	9% (6–13%)	3.40 (2.02–5.72)	3.39 (2.01–5.70)
Gallbladder	68	5	7% (3–16%)	2.81 (1.09–7.20)	2.96 (1.15–7.62)
Mesothelioma	75	4	5% (2–13%)	1.99 (0.71–5.61)	1.88 (0.66–5.31)
Lymphoma	698	33	5% (3–7%)	1.75 (1.14–2.69)	2.10 (1.37–3.23)
Vulval	73	3	4% (1–11%)	1.51 (0.46–4.94)	1.70 (0.52–5.60)
Lung	1875	77	4% (3–5%)	1.51 (1.08–2.12)	1.49 (1.07–2.09)
Melanoma	834	30	4% (3–5%)	1.32 (0.85–2.05)	1.69 (1.09–2.64)
Bladder	842	28	3% (2–5%)	1.22 (0.78–1.91)	1.16 (0.74–1.82)
Colorectal	2399	66	3% (2–3%)	Ref.	Ref.
Stomach	302	8	3% (1–5%)	0.96 (0.46–2.02)	0.94 (0.45–1.99)
Ovarian	394	10	3% (1–5%)	0.92 (0.47–1.81)	1.11 (0.56–2.20)
Laryngeal	121	3	2% (1–7%)	0.90 (0.28–2.90)	0.96 (0.30–3.12)
Oropharyngeal	213	5	2% (1–5%)	0.85 (0.34–2.13)	1.05 (0.42–2.64)
Small Intestine	53	1	2% (0.3–10%)	0.68 (0.09–4.99)	0.72 (0.10–5.28)
Pancreatic	370	6	2% (1–3%)	0.58 (0.25–1.35)	0.59 (0.26–1.38)
Endometrial	410	6	1% (1–3%)	0.52 (0.23–1.22)	0.61 (0.26–1.43)
Testicular	149	2	1% (0.4–5%)	0.48 (0.12–1.98)	1.05 (0.25–4.43)
Breast	2675	34	1% (1–2%)	0.46 (0.30–0.69)	0.59 (0.38–0.91)
Sarcoma	106	1	0.9% (0.2–5%)	0.34 (0.05–2.45)	0.43 (0.06–3.14)
Brain	215	2	0.9% (0.3–3%)	0.33 (0.08–1.36)	0.37 (0.09–1.54)
Oesophageal	566	3	0.5% (0.2–2%)	0.19 (0.06–0.60)	0.19 (0.06–0.60)
Cervical	125	0	0% (0–3%)	N/A	N/A

[a] adjusted for sex, age group, and cancer site
[b] joint Wald test p-value

For peer review only

Among the 520 incidentally diagnosed patients, a fifth (20%, 95% CI: 17–23%) were diagnosed with leukaemia, while other common cancer sites included lung (15%, 12–18%), colorectal (13%, 10–16%), and renal cancers (9%, 7–12%) (see Figure 3 and Supplementary Table 1). There were 9 other cancer sites represented amongst the incidentally diagnosed cancer patient population with 10 or more patients each.

Sensitivity analyses (using a broader definition of incidental diagnosis) identified a further 272 cases, increasing the overall estimate of incidental diagnosis to 6% (see Supplementary Table 2 and Supplementary Figure 2). There was weak evidence to support greater odds of incidental diagnosis among men versus women (adjusted OR (95% CI): 0.84 (0.71–1.00)), with otherwise similar patterns of variation by age group and cancer site as in the main analysis.

Routes to incidental cancer diagnosis

We identified several clinical scenarios preceding an incidental diagnosis of cancer based on information available for 345 patients (66% of all incidentally diagnosed patients). These are outlined in Table 2 and discussed in further detail below.

Many patients received an incidental cancer diagnosis as a result of a clinical encounter for a pre-existing chronic disease in primary or secondary care. This included routine blood or urine testing, as part of chronic disease (or related risk factor) management and monitoring, which revealed abnormalities that led to the diagnosis of unsuspected cancer. Some patients were diagnosed following blood or imaging investigations before/after elective surgery for unrelated indications with a small number of patients where tumours were identified during surgery. A small number of patients were diagnosed after blood or imaging investigations conducted as part of follow up for a pre-existing cancer (e.g. scans to ascertain stage at diagnosis of prostate cancer led to the diagnosis of a renal cancer).

Other cancer patients were diagnosed following the investigation of unrelated acute conditions or presenting symptom(s) unlikely to be related to the subsequent cancer diagnosis. Several of these cases were being investigated for another suspected cancer (e.g. a CT scan for a suspected pelvic cancer leading to the diagnosis of colorectal cancer) but in others the diagnosis was more serendipitous (e.g. breast lump found on examination for chest infection).

Table 2 Clinical scenarios preceding the incidental diagnosis of cancer

Clinical scenario	Description and examples
Monitoring or managing pre-existing chronic morbidity	Blood or imaging investigations as part of monitoring or management of a chronic morbidity<br>E.g. haematuria on dipstick urine testing [for diabetes] led to diagnosis of bladder cancer<br>E.g. annual blood tests for hypertension led to diagnosis of leukaemia
Before/after surgery	Blood or imaging investigations conducted before or after surgery, or more rarely, tumours identified during elective surgery for unrelated condition<br>E.g. pre-operative chest x-ray leading to diagnosis of lung cancer<br>E.g. microscopic haematuria noted pre-cataract operation leading to diagnosis of a urological cancer
Follow up of a pre-existing cancer	Blood or imaging investigations conducted as part of follow up for a pre-existing cancer

For peer review only

	E.g. scans to ascertain stage at diagnosis of prostate cancer leading to the diagnosis of a urological cancer
Investigation of unrelated acute or new condition or symptoms	Blood or imaging investigations for a new symptom or otherwise acute condition<br>E.g. an abdominal ultrasound scan for dyspepsia leading to the diagnosis of a urological cancer<br>E.g. abnormal result or irregular mole noted during health check

For peer review only

DISCUSSION

Principal findings

Around 1 in 25 cancer patients in our study population were diagnosed incidentally, with a preponderance among older patients, and patients with leukaemia, renal cancer, thyroid cancer, liver cancer, and multiple myeloma. Several clinical scenarios preceded incidental diagnosis including healthcare encounters due to previously known conditions and the investigation of acute or new conditions unrelated to cancer.

Strengths and limitations

Information on incidental status at diagnosis is not routinely recorded as part of cancer registration, healthcare records, other administrative databases, or patient experience surveys. A strength of our study is that it provides unique evidence about this less well documented diagnostic pathway of cancer, among a large and representative incident cohort of cancer patients. Furthermore, we have identified incidentally diagnosed patients using a novel approach, based on the triangulation of information regarding symptom status, test results, and explicit mention of incidental diagnosis (Figure 1).

Nevertheless, interpretation of the findings should be mindful of the secondary nature of our analysis, and the period of data collection. Information on symptoms (or their absence) was based on those recorded in primary care; patients found to be asymptomatic by auditors may have had symptoms that were either not declared during the consultation, or else not recorded in their records [19,20]. In order to reduce the risk of the resulting bias on analyses, our definition of incidentally diagnosed cancer was deliberately conservative, designed to maximise specificity and reduce the likelihood of patients being mistakenly identified as incidental diagnoses. However, this may have led to the under-estimation of cases; our sensitivity analysis (based on a less conservative definition, see Figure 1) indicates that an additional 2% of the study population may have been incidentally diagnosed (Supplementary Table 2). Although the true estimates of incidental diagnosis may be higher than those reported, this is unlikely to have biased patterns of variation by cancer site and patient characteristics.

Comparison with existing literature

Literature examining incidentally diagnosed cancer is limited, although some evidence may be gleaned from studies on incidental findings detected in the context of research studies. Estimates of clinically important incidental findings (including cancer but also other diseases) vary substantially depending on imaging field (whole body, or specific organ) and modality however, and participants of research studies are unlikely to be representative of the general population [21,22].

Though we were unable to examine potential overdiagnosis, we identified notable proportions of incidentally diagnosed patients with thyroid and renal cancer, and melanoma patients. This is consistent with prior evidence indicating potential overdiagnosis of these cancers [23–26]. A few studies have examined clinical scenarios that result in incidental diagnosis of individual cancer sites such as melanoma, lung cancer, and renal cancer [17,27–29]. A study examining self-reported symptoms of haematological cancer patients found that a third of patients did not report any symptoms before diagnosis, with chronic lymphocytic leukaemia patients being particularly prone to being diagnosed incidentally, for example through blood tests at routine healthcare encounters [30].

For peer review only

Our findings are in agreement with these studies, but additionally suggest that incidental diagnosis occurs across a range of common and rarer cancers.

Implications

Our findings indicate that a substantial proportion of cancer patients are diagnosed with cancer incidentally, without having presented with symptoms related to the subsequent diagnosis. An incidental cancer diagnosis could represent fortuitous early diagnosis of an invasive tumour, and therefore be of clinical benefit for a proportion of patients. However it could also represent overdiagnosis, which could lead to considerable psychological morbidity and unnecessary treatment.

We identified several clinical scenarios that resulted in the incidental diagnosis of cancer; their frequency is likely to be affected by system level factors such as approaches to chronic disease monitoring, incentives and thresholds for investigation, availability of imaging services, and rates of elective surgery [31,32]. Given increasing levels of multi-morbidity and an ageing population, there is progressively greater use of blood-based testing and imaging studies, which could lead to a greater proportion of patients being diagnosed incidentally particularly for certain cancer types such as leukaemia [10]. Relatedly, incidental diagnosis of cancer occurred during investigation or follow up of a pre-existing (unrelated) tumour in a small number of patients. As the survival of patients with cancer continues to improve, this could also become a more prevalent route to incidental diagnosis [33]. Further examination of incidentally diagnosed cancer among more contemporary populations would be helpful in this regard.

Conclusions

In conclusion, we have provided evidence about the frequency and common scenarios leading to incidental diagnosis of cancer. Our findings indicate that this is likely to affect around one in 25 cancer patients and calls for further research establishing the prognostic, psychosocial and economic implications of incidentally diagnosed cancer.

For peer review only

REFERENCES

1 Elliss-Brookes L, McPhail S, Ives A, *et al.* Routes to diagnosis for cancer - determining the patient journey using multiple routine data sets. *Br J Cancer* 2012;**107**:1220–6. doi:10.1038/bjc.2012.408

2 Jensen H, Tørring ML, Olesen F, *et al.* Cancer suspicion in general practice, urgent referral and time to diagnosis: a population-based GP survey and registry study. *BMC Cancer* 2014;**14**:636. doi:10.1186/1471-2407-14-636

3 Lumbreras B, Donat L, Hernández-Aguado I. Incidental findings in imaging diagnostic tests: a systematic review. *Br J Radiol* 2010;**83**:276–89. doi:10.1259/bjr/98067945

4 Kroczek EK, Wieners G, Steffen I, *et al.* Non-traumatic incidental findings in patients undergoing whole-body computed tomography at initial emergency admission. *Emerg Med J* 2017;:emermed-2016-205722. doi:10.1136/emermed-2016-205722

5 O'Sullivan JW, Muntinga T, Grigg S, *et al.* Prevalence and outcomes of incidental imaging findings: umbrella review. *BMJ* 2018;:k2387. doi:10.1136/bmj.k2387

6 Maskell G. Think before you scan. *Bmj* 2018;**3754**:k3754. doi:10.1136/bmj.k3754

7 NHS. NHS Health Check. 2017.http://www.healthcheck.nhs.uk/

8 NHS Digital. Quality and Outcomes Framework. Prim. Care. 2016.http://content.digital.nhs.uk/QOF

9 Treadwell J, McCartney M. Overdiagnosis and overtreatment: generalists--it's time for a grassroots revolution. *Br J Gen Pract* 2016;**66**:116–7. doi:10.3399/bjgp16X683881

10 O'Sullivan JW, Stevens S, Hobbs FDR, *et al.* Temporal trends in use of tests in UK primary care, 2000-15: retrospective analysis of 250 million tests. *Bmj* 2018;:k4666. doi:10.1136/bmj.k4666

11 Esserman LJ, Thompson IM, Reid B, *et al.* Addressing overdiagnosis and overtreatment in cancer: A prescription for change. *Lancet Oncol* 2014;**15**:e234–42. doi:10.1016/S1470-2045(13)70598-9

12 Jenniskens K, de Groot JAH, Reitsma JB, *et al.* Overdiagnosis across medical disciplines: a scoping review. *BMJ Open* 2017;**7**:e018448. doi:10.1136/bmjopen-2017-018448

13 Davies L, Petitti DB, Martin L, *et al.* Defining, Estimating, and Communicating Overdiagnosis in Cancer Screening. *Ann Intern Med* 2018;**169**:36. doi:10.7326/M18-0694

14 Rubin GP, McPhail S, Elliot K, *et al.* National Audit of Cancer Diagnosis in Primary Care. London: 2011. http://www.rcgp.org.uk/policy/rcgp-policy-areas/national-audit-of-cancer-diagnosis-in-primary-care.aspx

15 Lyratzopoulos G, Abel GA, McPhail S, *et al.* Gender inequalities in the promptness of diagnosis of bladder and renal cancer after symptomatic presentation: evidence from secondary analysis of an English primary care audit survey. *BMJ Open* 2013;**3**:e002861. doi:10.1136/bmjopen-2013-002861

16 Davies L, Ouellette M, Hunter M, *et al.* The increasing incidence of small thyroid cancers: Where are the cases coming from? *Laryngoscope* 2010;**120**:2446–51. doi:10.1002/lary.21076

17 Kocher F, Lunger F, Seeber A, *et al.* Incidental Diagnosis of Asymptomatic Non-Small-Cell Lung Cancer: A Registry-Based Analysis. *Clin Lung Cancer* 2016;**17**:62–7. doi:10.1016/j.cllc.2015.08.006

For peer review only

18 NICE. Prostate cancer : diagnosis and management (CG175). Published Online First: 2014.https://www.nice.org.uk/guidance/cg175

19 Larsen MB, Hansen RP, Sokolowski I, *et al.* Agreement between patient-reported and doctor-reported patient intervals and date of first symptom presentation in cancer diagnosis – A population-based questionnaire study. *Cancer Epidemiol* 2014;**38**:100–5. doi:10.1016/j.canep.2013.10.006

20 Leiva A, Esteva M, Llobera J, *et al.* Time to diagnosis and stage of symptomatic colorectal cancer determined by three different sources of information: A population based retrospective study. *Cancer Epidemiol* 2017;**47**:48–55. doi:10.1016/j.canep.2016.10.021

21 The Royal College of Radiologists. Management of Incidental Findings Detected During Research Imaging. 2011. papers3://publication/uuid/627D4627-FE73-472B-BD26-9FD0F38FC547

22 Booth TC, Jackson A, Wardlaw JM, *et al.* Incidental findings found in 'healthy' volunteers during imaging performed for research: Current legal and ethical implications. *Br J Radiol* 2010;**83**:456–65. doi:10.1259/bjr/15877332

23 Weyers W. The 'epidemic' of melanoma between under- and overdiagnosis. *J Cutan Pathol* 2012;**39**:9–16. doi:10.1111/j.1600-0560.2011.01831.x

24 Welch HG, Black WC. Overdiagnosis in cancer. *J Natl Cancer Inst* 2010;**102**:605–13. doi:10.1093/jnci/djq099

25 Moynihan R, Doust J, Henry D. Preventing overdiagnosis: how to stop harming the healthy. *Bmj* 2012;**344**:e3502–e3502. doi:10.1136/bmj.e3502

26 Ahn HS, Kim HJ, Welch HG. Korea's Thyroid-Cancer "Epidemic" — Screening and Overdiagnosis. *N Engl J Med* 2014;**371**:1765–7. doi:10.1056/NEJMp1409841

27 Cufari ME, Proli C, Phull M, *et al.* Increasing incidence of non-smoking lung cancer: presentation of patients with early disease to a tertiary institution in the UK. *Lung Cancer* 2016;**91**:S17–8. doi:10.1016/S0169-5002(16)30066-6

28 Avilés-Izquierdo JA, Molina-López I, Rodríguez-Lomba E, *et al.* Who detects melanoma? Impact of detection patterns on characteristics and prognosis of patients with melanoma. *J Am Acad Dermatol* 2016;:1–8. doi:10.1016/j.jaad.2016.07.009

29 Hofbauer SL, de Martino M, Seemann C, *et al.* Associations between presenting symptoms, clinicopathological parameters, and prognosis in a contemporary series of patients with renal cell carcinoma. *Korean J Urol* 2014;**55**:505–10. doi:10.4111/kju.2014.55.8.505

30 Howell DA, Smith AG, Jack A, *et al.* Time-to-diagnosis and symptoms of myeloma, lymphomas and leukaemias: a report from the Haematological Malignancy Research Network. *BMC Blood Disord* 2013;**13**:9. doi:10.1186/2052-1839-13-9

31 Pollack CE, Soulos PR, Herrin J, *et al.* The Impact of Social Contagion on Physician Adoption of Advanced Imaging Tests in Breast Cancer. *J Natl Cancer Inst* 2017;**109**:1–8. doi:10.1093/jnci/djw330

32 Barraclough K. New NICE guidance on referral for cancer. *BMJ* 2015;**351**:h3640. doi:10.1136/bmj.h3640

33 Murphy CC, Gerber DE, Pruitt SL. Prevalence of Prior Cancer Among Persons Newly Diagnosed With Cancer. *JAMA Oncol* 2017;**75390**:1–4. doi:10.1001/jamaoncol.2017.3605

For peer review only

For peer review only

Additional information

Acknowledgements

We are grateful to all general practitioners and healthcare professionals that were involved in the collection and submission of anonymous data to the audit, and to the respective Cancer Networks, the Royal College of General Practitioners, the former National Cancer Action Team and the former National Clinical Intelligence Network (NCIN) of Public Health England (PHE) for supporting the audit.

Competing interests

The authors have declared no competing interests.

Funding statement

This work was supported by the UK Department of Health as part of the programme of the Policy Research Unit in Cancer Awareness, Screening and Early Diagnosis [grant number no. 106/0001]. The Policy Research Unit in Cancer Awareness, Screening, and Early Diagnosis is a collaboration between researchers from seven institutions (Queen Mary University of London, University College London, King's College London, London School of Hygiene and Tropical Medicine, Hull York Medical School, Durham University and Peninsula Medical School/University of Exeter). GL is supported by Cancer Research UK Clinician Advanced Scientist Fellowship [grant number: C18081/A18180]. The views expressed are those of the authors and not necessarily those of the Department of Health or Cancer Research UK. The funders of the study had no role in the study design, data analysis, data interpretation, or writing of the report. The corresponding author had full access to all the data in the study and had final responsibility for the decision to submit for publication.

Authorship contribution

MMK, GPR, and GL conceived the study. MMK conducted all statistical analyses with assistance from GL. MMK wrote the first draft of the manuscript, and prepared the tables and figures, supervised by GL. MMK, GPR, SMcP, and GL contributed to the interpretation of the results, revised the manuscript and approved the final version of the manuscript.

Data sharing agreement

The data used for our analysis is available from the National Cancer Registration and Analysis Service. Enquiries for data access can be made to Public Health England's Office for Data Release (odr@phe.gov.uk).

Figure/Table legends

Figure 1 Visualisation of characteristics used to identify cases of incidentally diagnosed cancer. Areas shaded dark blue represent cases included in the main analysis, while areas in light blue indicate additional cases included in the sensitivity analysis.

Figure 2 Odds ratios of incidental versus symptomatic diagnosis of cancer by cancer site (n=13,810; reference group: colorectal cancer). NB there is no odds ratio for ovarian cervical cancer as there were no incidentally diagnosed cases of cervical cancer.

Figure 3 Commonly diagnosed cancer sites among the incidental cancer patient population; see Supplementary Table 1 for frequencies

For peer review only

Table 1 Characteristics of incidental cancer patients versus non-incidental cancer patients, and crude/adjusted odds ratios of incidental status (n=13,810)

Table 2 Clinical scenarios preceding the incidental diagnosis of cancer

For peer review only

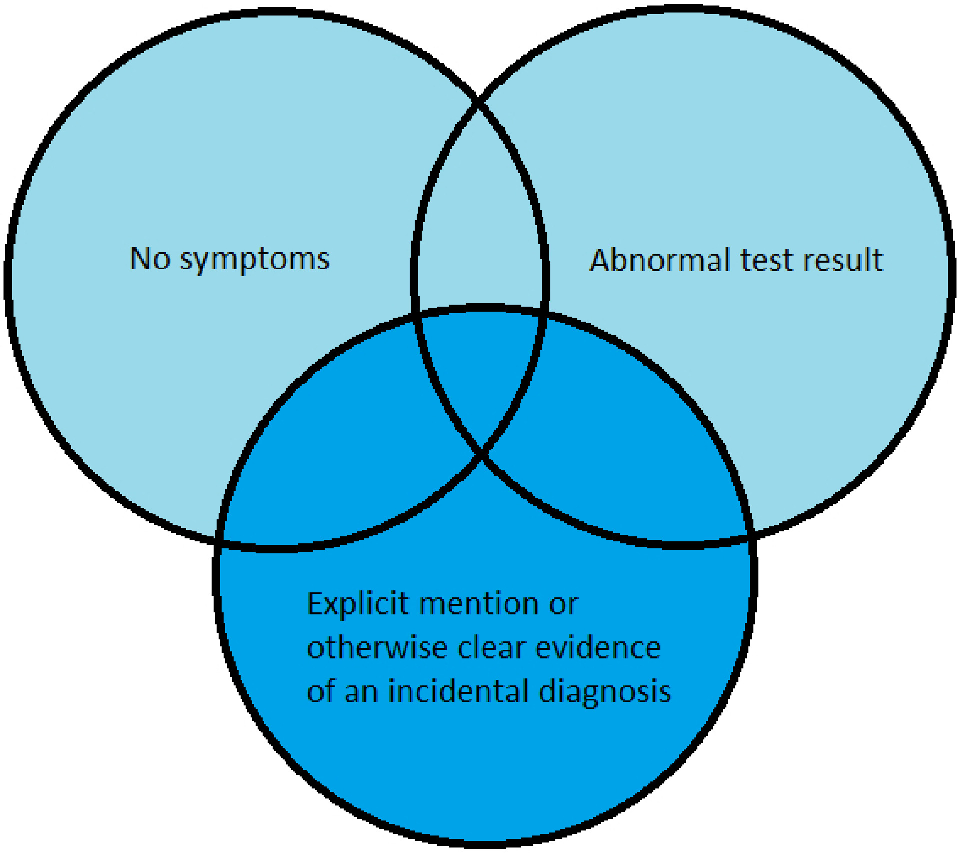

Figure 1: Visualisation of characteristics used to identify cases of incidentally diagnosed cancer. Areas shaded dark blue represent cases included in the main analysis, while areas in light blue indicate additional cases included in the sensitivity analysis.

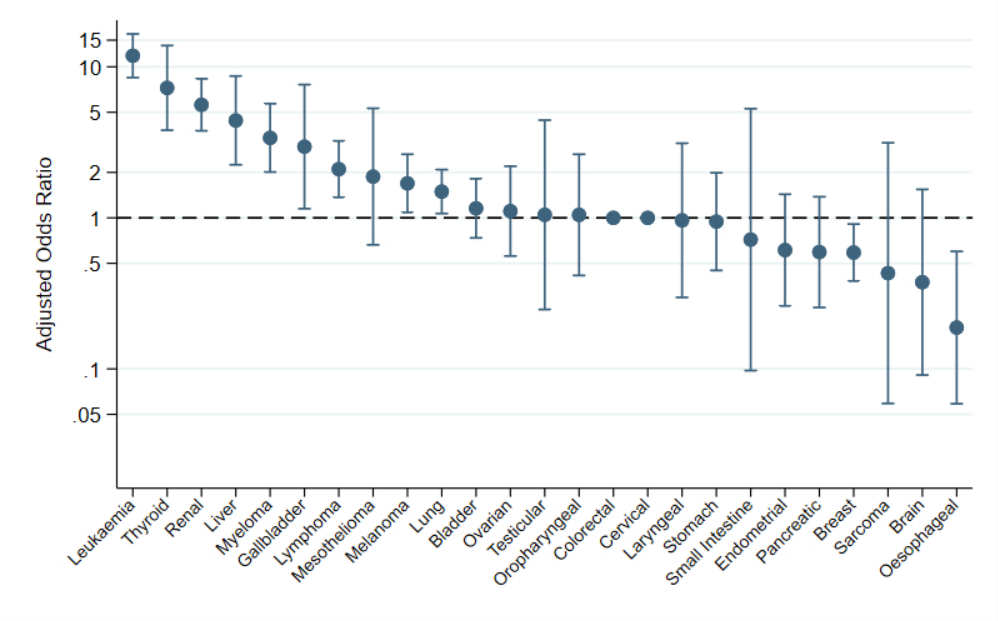

Figure 2: Odds ratios of incidental versus symptomatic diagnosis of cancer by cancer site (n=13,810; reference group: colorectal cancer). NB there is no odds ratio for cervical cancer as there were no incidentally diagnosed cases of cervical cancer.

282x176mm (72 x 72 DPI)

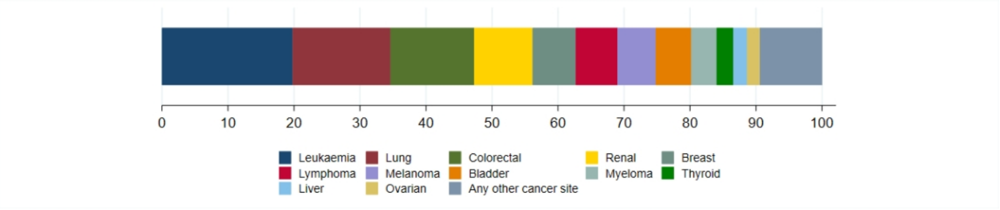

Figure 3: Commonly diagnosed cancer sites among the incidental cancer patient population; see Supplementary Table 1 for frequencies

423x88mm (72 x 72 DPI)

Supplementary information for "*Incidentally diagnosed cancer and commonly preceding clinical scenarios: a cross-sectional descriptive analysis of English audit data*" Koo et al., 2019

Table of contents

- Supplementary Figure 1: Flow chart describing sample derivation for main analysis
- Supplementary Table 1: Cancer site case-mix of incidentally diagnosed cancer patients
- Supplementary Table 2 [Sensitivity analysis]: Characteristics of incidental cancer patients versus non-incidental cancer patients, and crude/adjusted odds ratios of incidental status (n=14,082)
- Supplementary Figure 2 [Sensitivity analysis]: Odds ratios of incidental versus symptomatic diagnosis of cancer by cancer site (n=14,082; reference group: colorectal cancer)

For peer review only

Supplementary files for Koo et al., 2019

Supplementary Figure 1: Flow chart describing sample derivation for main analysis

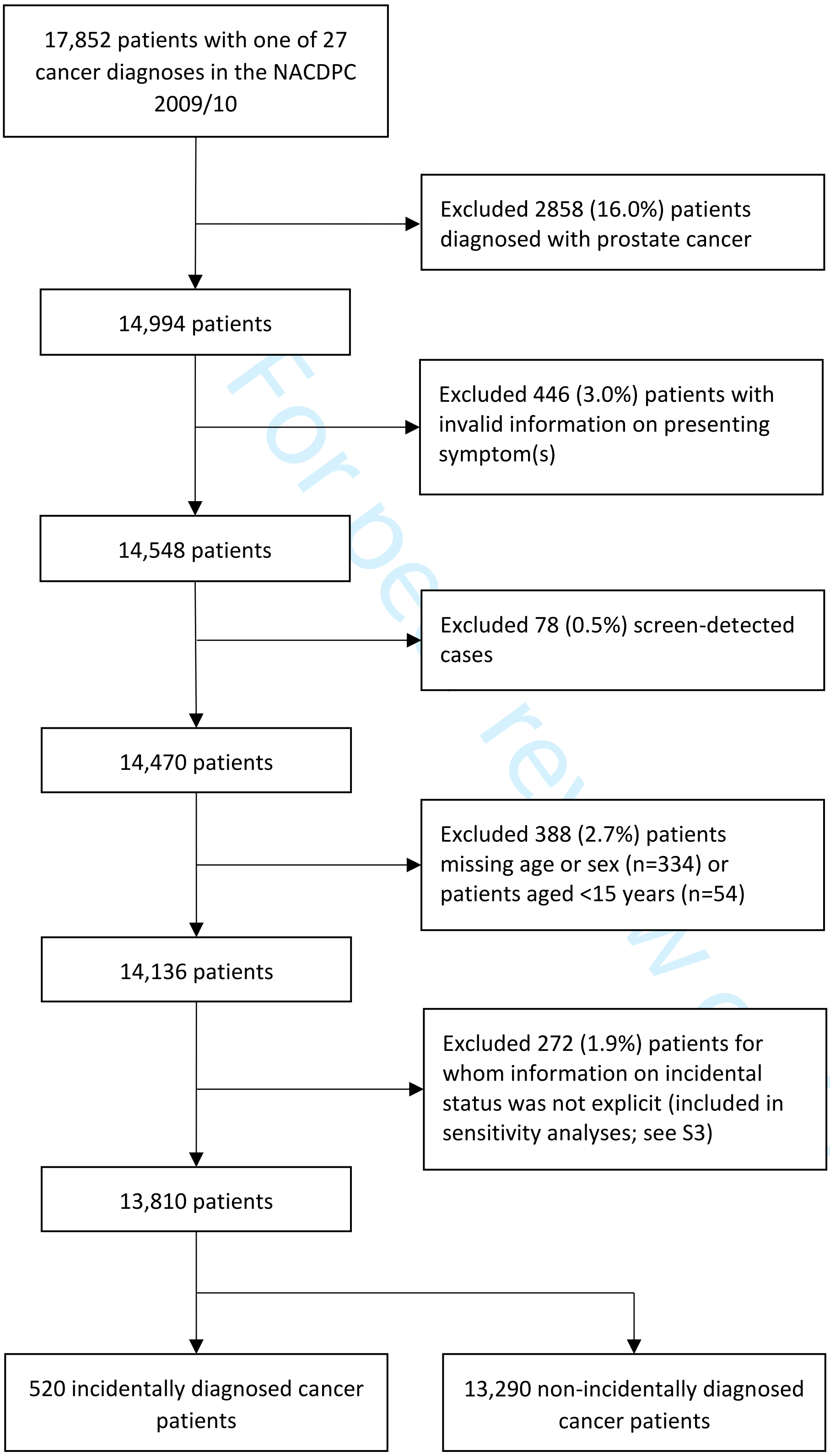

Supplementary Table 1: Cancer site case-mix of incidentally diagnosed cancer patients

Cancer	N	% (95% CI)
Leukaemia	103	20% (17–23%)
Lung	77	15% (12–18%)
Colorectal	66	13% (10–16%)
Renal	46	9% (7–12%)
Breast	34	7% (5–9%)
Lymphoma	33	6% (5–9%)
Melanoma	30	6% (4–8%)
Bladder	28	5% (4–8%)
Myeloma	20	4% (3–6%)
Thyroid	13	3% (1–4%)
Liver	11	2.1% (1.2–3.7%)
Ovarian	10	1.9% (1.0–3.5%)
Stomach	8	1.5% (0.8–3.0%)
Endometrial	6	1.2% (0.5–2.5%)
Pancreatic	6	1.2% (0.5–2.5%)
Gallbladder	5	1.0% (0.4–2.2%)
Oropharyngeal	5	1.0% (0.4–2.2%)
Mesothelioma	4	0.8% (0.3–2.0%)
Laryngeal	3	0.6% (0.2–1.7%)
Oesophageal	3	0.6% (0.2–1.7%)
Vulval	3	0.6% (0.2–1.7%)
Brain	2	0.4% (0.1–1.4%)
Testicular	2	0.4% (0.1–1.4%)
Sarcoma	1	0.2% (0.03–1.1%)
Small Intestine	1	0.2% (0.03–1.1%)
Total	520	100%

* Proportion of patients with each cancer site, of the total incidentally diagnosed population (n=520)

For peer review only

Supplementary files for Koo et al., 2019

Supplementary Table 2 [Sensitivity analysis]: Characteristics of incidental cancer patients versus non-incidental cancer patients, and crude/adjusted odds ratios of incidental status (n=14,082)

	Total	Incidental		Crude	Adjusted[a]
	N	n	% (95% CI)	OR (95% CI)	OR (95% CI)
Total	14082	792	6% (5–6%)	-	-
**Sex**				**0.001[b]**	**0.045[b]**
Men	5983	422	7% (6–8%)	Ref.	Ref.
Women	8099	370	5% (4–5%)	0.63 (0.55–0.73)	0.84 (0.71–1.00)
**Age group**				**<0.001[b]**	**<0.001[b]**
15–49 years	2089	48	2% (2–3%)	0.36 (0.26–0.50)	0.36 (0.26–0.51)
50–59 years	2080	95	5% (4–6%)	0.73 (0.57–0.94)	0.75 (0.58–0.98)
60–69 years	3264	200	6% (5–7%)	Ref.	Ref.
70–79 years	3739	253	7% (6–8%)	1.11 (0.92–1.35)	1.08 (0.89–1.33)
80+ years	2910	196	7% (6–8%)	1.11 (0.90–1.36)	1.17 (0.94–1.45)
**Cancer site**				**<0.001[b]**	**<0.001[b]**
Leukaemia	511	164	32% (28–36%)	11.25 (8.55–14.81)	12.48 (9.46–16.48)
Liver	116	24	21% (14–29%)	6.21 (3.79–10.16)	6.28 (3.82–10.32)
Renal	373	63	17% (13–21%)	4.84 (3.45–6.78)	5.12 (3.64–7.19)
Thyroid	113	16	14% (9–22%)	3.93 (2.23–6.92)	5.93 (3.31–10.60)
Myeloma	241	33	14% (10–19%)	3.78 (2.48–5.74)	3.74 (2.46–5.69)
Gallbladder	68	5	7% (3–16%)	1.89 (0.74–4.80)	2.00 (0.78–5.09)
Bladder	869	55	6% (5–8%)	1.61 (1.15–2.26)	1.52 (1.08–2.14)
Lung	1913	115	6% (5–7%)	1.52 (1.15–2.01)	1.50 (1.14–1.97)
Lymphoma	704	39	6% (4–7%)	1.40 (0.95–2.04)	1.64 (1.12–2.41)
Mesothelioma	75	4	5% (2–13%)	1.34 (0.48–3.75)	1.23 (0.44–3.45)
Melanoma	839	35	4% (3–6%)	1.04 (0.70–1.54)	1.30 (0.88–1.94)
Vulval	73	3	4% (1–11%)	1.02 (0.32–3.30)	1.19 (0.37–3.87)
Colorectal	2431	98	4% (3–5%)	Ref.	Ref.
Ovarian	398	14	4% (2–6%)	0.87 (0.49–1.54)	1.05 (0.59–1.88)
Stomach	303	9	3% (2–6%)	0.73 (0.36–1.46)	0.72 (0.36–1.44)
Breast	2717	76	3% (2–3%)	0.69 (0.51–0.93)	0.91 (0.66–1.25)
Pancreatic	374	10	3% (1–5%)	0.65 (0.34–1.27)	0.66 (0.34–1.28)
Laryngeal	121	3	2% (1–7%)	0.61 (0.19–1.94)	0.62 (0.19–1.98)
Oropharyngeal	213	5	2% (1–5%)	0.57 (0.23–1.42)	0.68 (0.27–1.69)
Endometrial	413	9	2% (1–4%)	0.53 (0.27–1.06)	0.62 (0.31–1.24)
Small Intestine	53	1	2% (–10%)	0.46 (0.06–3.35)	0.48 (0.07–3.55)
Sarcoma	107	2	2% (1–7%)	0.45 (0.11–1.86)	0.57 (0.14–2.36)
Testicular	149	2	1% (–5%)	0.32 (0.08–1.33)	0.66 (0.16–2.76)
Brain	215	2	1% (–3%)	0.22 (0.05–0.91)	0.25 (0.06–1.02)
Cervical	126	1	1% (–4%)	0.19 (0.03–1.38)	0.29 (0.04–2.09)
Oesophageal	567	4	1% (–2%)	0.17 (0.06–0.46)	0.17 (0.06–0.45)

[a] adjusted for sex, age group, and cancer site
[b] joint Wald test p-value

For peer review only

Supplementary files for Koo et al., 2019

Supplementary Figure 2 [Sensitivity analysis]: Odds ratios of incidental versus symptomatic diagnosis of cancer by cancer site (n=14,082; reference group: colorectal cancer)

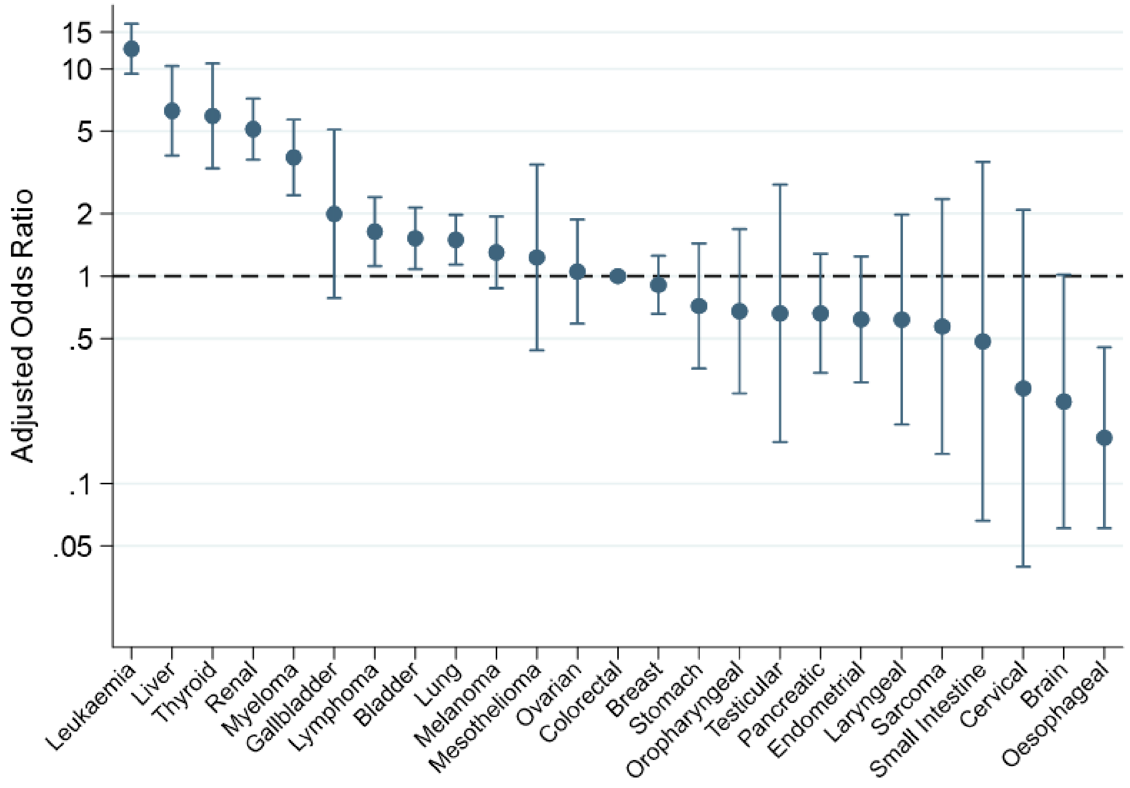

STROBE Statement—Checklist of items that should be included in reports of ***cross-sectional studies***

NB we have added bold text to the "Recommendation" column indicating the presence of each item recommended by the STROBE statement in the submitted BMJ Open manuscript ID bmjopen-2018-028362

	Item No	Recommendation
**Title and abstract**	1	(*a*) Indicate the study's design with a commonly used term in the title or the abstract<br>**See title, first page**
		(*b*) Provide in the abstract an informative and balanced summary of what was done and what was found<br>**See Abstract**
**Introduction**		
Background/rationale	2	Explain the scientific background and rationale for the investigation being reported<br>**See Introduction, first/second paragraphs**
Objectives	3	State specific objectives, including any prespecified hypotheses<br>**See Introduction, last paragraph**
**Methods**		
Study design	4	Present key elements of study design early in the paper<br>**See Methods: Study design and population subsection**
Setting	5	Describe the setting, locations, and relevant dates, including periods of recruitment, exposure, follow-up, and data collection<br>**See Methods: Study design and population subsection**
Participants	6	(*a*) Give the eligibility criteria, and the sources and methods of selection of participants<br>**See Methods: Study design and population subsection**
Variables	7	Clearly define all outcomes, exposures, predictors, potential confounders, and effect modifiers. Give diagnostic criteria, if applicable<br>**See Methods: Definition and identification of cases, and Data analysis subsections**
Data sources/ measurement	8*	For each variable of interest, give sources of data and details of methods of assessment (measurement). Describe comparability of assessment methods if there is more than one group<br>**See Methods: Study design and population; and Methods: Definition and identification of cases subsections**
Bias	9	Describe any efforts to address potential sources of bias<br>**See Methods: Data analysis subsection**
Study size	10	Explain how the study size was arrived at<br>**See Methods: Definition and identification of cases subsection, last sentence, and Figure S1 in Supplementary materials**
Quantitative variables	11	Explain how quantitative variables were handled in the analyses. If applicable, describe which groupings were chosen and why<br>**See Methods: Data analysis subsection**
Statistical methods	12	(*a*) Describe all statistical methods, including those used to control for confounding<br>**See Methods: Data analysis subsection**
		(*b*) Describe any methods used to examine subgroups and interactions<br>**N/A**
		(*c*) Explain how missing data were addressed

For peer review only - http://bmjopen.bmj.com/site/about/guidelines.xhtml

		**See Discussion: Strengths and limitations subsection**
		(*d*) If applicable, describe analytical methods taking account of sampling strategy **N/A**
		(*e*) Describe any sensitivity analyses **See Methods: Sensitivity analysis subsection**
**Results**		
Participants	13*	(a) Report numbers of individuals at each stage of study—eg numbers potentially eligible, examined for eligibility, confirmed eligible, included in the study, completing follow-up, and analysed **See Methods: Definition and identification of cases subsection and Figure S1 in Supplementary materials**
		(b) Give reasons for non-participation at each stage **See Methods: Definition and identification of cases subsection**
		(c) Consider use of a flow diagram **See Figure S1 in Supplementary materials**
Descriptive data	14*	(a) Give characteristics of study participants (eg demographic, clinical, social) and information on exposures and potential confounders **See Results: Incidentally diagnosed cancer patients subsection and Table 1, first 3 columns excluding the left-most column**
		(b) Indicate number of participants with missing data for each variable of interest **N/A**
Outcome data	15*	Report numbers of outcome events or summary measures **See Results: Incidentally diagnosed cancer patients subsection and Table 1, first 3 columns excluding the left-most column**
Main results	16	(*a*) Give unadjusted estimates and, if applicable, confounder-adjusted estimates and their precision (eg, 95% confidence interval). Make clear which confounders were adjusted for and why they were included **See Results: Incidentally diagnosed cancer patients subsection, Table 1, Figure 3, Table S2**
		(*b*) Report category boundaries when continuous variables were categorized **N/A**
		(*c*) If relevant, consider translating estimates of relative risk into absolute risk for a meaningful time period **N/A**
Other analyses	17	Report other analyses done—eg analyses of subgroups and interactions, and sensitivity analyses **See Results: Incidentally diagnosed cancer patients subsection paragraph 3 for details of the sensitivity analysis**
**Discussion**		
Key results	18	Summarise key results with reference to study objectives **See Discussion: Principal findings subsection**
Limitations	19	Discuss limitations of the study, taking into account sources of potential bias or imprecision. Discuss both direction and magnitude of any potential bias **See Discussion: strengths and limitations subsection**
Interpretation	20	Give a cautious overall interpretation of results considering objectives, limitations, multiplicity of analyses, results from similar studies, and other relevant evidence **See Discussion: strengths and limitations and Discussion: comparison with**

For peer review only - http://bmjopen.bmj.com/site/about/guidelines.xhtml

		**existing literature subsections**
Generalisability	21	Discuss the generalisability (external validity) of the study results<br>**See Discussion: strengths and limitations subsection**
**Other information**		
Funding	22	Give the source of funding and the role of the funders for the present study and, if applicable, for the original study on which the present article is based<br>**See Additional information: Funding statement subsection**

*Give information separately for exposed and unexposed groups.

Note: An Explanation and Elaboration article discusses each checklist item and gives methodological background and published examples of transparent reporting. The STROBE checklist is best used in conjunction with this article (freely available on the Web sites of PLoS Medicine at http://www.plosmedicine.org/, Annals of Internal Medicine at http://www.annals.org/, and Epidemiology at http://www.epidem.com/). Information on the STROBE Initiative is available at www.strobe-statement.org.

For peer review only

BMJ Open

Incidentally diagnosed cancer and commonly preceding clinical scenarios: a cross-sectional descriptive analysis of English audit data

Journal:	*BMJ Open*
Manuscript ID	bmjopen-2018-028362.R2
Article Type:	Research
Date Submitted by the Author:	11-Jun-2019
Complete List of Authors:	Koo, Minjoung; UCL, Behavioural Science and Health<br>Rubin, Greg; Royal Victoria Infirmary, Institute of Health and Society, Newcastle University<br>McPhail, Sean; Public Health England, NCRAS; UCL, Behavioural Science and Health<br>Lyratzopoulos, Georgios ; UCL, Behavioural Science and Health; Public Health England, NCRAS
**Primary Subject Heading**:	Health services research
Secondary Subject Heading:	Radiology and imaging, Oncology, Diagnostics, General practice / Family practice, Haematology (incl blood transfusion)
Keywords:	PRIMARY CARE, RADIOLOGY & IMAGING, PUBLIC HEALTH, HEALTH SERVICES ADMINISTRATION & MANAGEMENT, ONCOLOGY, PATHOLOGY

SCHOLARONE™
Manuscripts

Incidentally diagnosed cancer and commonly preceding clinical scenarios: a cross-sectional descriptive analysis of English audit data

Minjoung Monica Koo PhD [a], Greg P Rubin MD [b], Sean McPhail PhD [a,c], & Georgios Lyratzopoulos MD [a,c]

Affiliations:
[a] University College London, Gower street, London WC1E 6BT, UK
[b] Institute of Health and Society, Newcastle University, Sir James Spence Institute, Royal Victoria Infirmary, Newcastle upon Tyne, NE1 4LP, UK
[c] National Cancer Registration and Analysis Service, Public Health England, Wellington House, 133-155 Waterloo Road, London, SE1 8UG, UK

Corresponding Author:
Dr Minjoung Monica Koo (monica.koo.14@ucl.ac.uk)

Funding statement:
This work was supported by a grant from the UK Department of Health [grant number no. 106/0001], as part of the program of the Policy Research Unit in Cancer Awareness, Screening and Early Diagnosis. The Policy Research Unit in Cancer Awareness, Screening, and Early Diagnosis receives funding for a research program from the Department of Health Policy Research Programme. It is a collaboration between researchers from seven institutions (Queen Mary University of London, University College London, King's College London, London School of Hygiene and Tropical Medicine, Hull York Medical School, Durham University, and Peninsula Medical School/University of Exeter). GL is supported by a Cancer Research UK Advanced Clinician Scientist Fellowship [grant number: C18081/A18180]. The views expressed are those of the authors and not necessarily those of the Department of Health or Cancer Research UK.

Competing interests:
None declared

Abstract: 270
Main text: 2150
References: 33
Tables: 2
Figures: 3

For peer review only

ABSTRACT

Objectives

Cancer can be diagnosed in the absence of tumour-related symptoms, but little is known about the frequency and circumstances preceding such diagnoses outside participation in screening programmes. We aimed to examine incidentally diagnosed cancer among a cohort of cancer patients diagnosed in England.

Design

Cross-sectional study of national primary care audit data on incident cancer patients.

Setting

We analysed free-text information on the presenting features of cancer patients aged 15 or older included in the English National Audit of Cancer Diagnosis in Primary Care (NACDPC) (2009-10). Patients with screen-detected cancers and those diagnosed with prostate cancer were excluded. We examined the odds of incidental cancer diagnosis by patient characteristics and cancer site using logistic regression, and described clinical scenarios leading to incidental diagnosis.

Results

Among the studied cancer patient population (n=13,810), 520 (4%) patients were diagnosed incidentally. The odds of incidental cancer diagnosis increased with age ($p<0.001$), with no difference between men and women after adjustment. Incidental diagnosis was most common among patients with leukaemia (23%), renal (13%) and thyroid cancer (12%), and least common among patients with brain (0.9%), oesophageal (0.5%), and cervical cancer (no cases diagnosed incidentally). Variation in odds of incidental diagnosis by cancer site remained after adjusting for age group and sex.

Incidental diagnoses were commonly preceded by a range of clinical scenarios across primary and secondary care. These included the monitoring or management of pre-existing conditions, routine testing before or after elective surgery, and the investigation of unrelated acute or new conditions.

Conclusions

One in 25 patients with cancer in our population-based cohort were diagnosed incidentally, through different mechanisms across primary and secondary care settings. The epidemiological, clinical, psychological, and economic implications of this diagnostic phenomenon merit further investigation.

Strengths and limitations of this study

- The findings are based on a unique large population-based cohort of individuals diagnosed with a range of cancers with detailed characterisation of their presenting features
- Diagnostic status (incidental or non-incidental) was identified using free-text information provided by primary care physicians based on primary care records
- We describe common mechanisms of incidental diagnosis beyond a single modality or cancer site with a high level of detail
- We were unable to examine differences in clinical outcome between incidental and non-incidentally diagnosed cancer patients

For peer review only

INTRODUCTION

Cancer is most often diagnosed following presentation with symptoms likely caused by the malignancy [1,2]. However, some patients are diagnosed with cancer incidentally, in the absence of symptoms that could not plausibly be related to the tumour and outside formal cancer screening or surveillance activity. The use of imaging technologies (including x-ray, CT, MRI, and PET scans) is one of the commonly described routes to incidental diagnosis of different diseases, including cancer [3–6]. Chronic disease management involving periodic routine blood or urine testing are increasingly used in primary care and may represent another common pathway to incidental diagnosis [7–10]. Nonetheless, evidence regarding the frequency of such incidental diagnoses is currently limited.

Since incidental cancer diagnoses are characterised by the absence of tumour related symptoms, it is plausible that this may represent overdiagnosis in some patients, whereby the detected cancer would not have otherwise caused symptoms in the patient's lifetime [11]. Concerns about overdiagnosis thus far have largely focused on screening-detected cancers (e.g. breast cancer), but it may be also occurring in other contexts [12,13]. Ahead of considering the clinical, psychological, or economic consequences associated with incidental diagnosis (including the potential for overdiagnosis), we need to address gaps in knowledge about the frequency and characteristics of incidentally diagnosed cancer.

We therefore aimed to examine the frequency of incidental diagnosis among an incident cohort of cancer patients; compare the characteristics of incidentally vs non-incidentally diagnosed patients; and examine common pathways and mechanisms likely to lead to incidental diagnosis of cancer using a unique data source relating to a national quality improvement initiative in England.

For peer review only

METHODS

Study design and population

We analysed cross-sectional data collected as part of the English National Audit of Cancer Diagnosis in Primary Care (NACDPC) [14]. Briefly, health professionals from 1,170 participating general practices (representing 14% of practices in England) provided information on the diagnostic pathway for a consecutive sample of patients diagnosed with cancer during April 2009–2010. Participating practices were comparable to non-participating practices in (former) respective Cancer Networks, and the patient population was broadly representative of the contemporary national incident cancer patient cohort [14,15]. Unique to this audit, clinicians participating in the NACDPC provided extensive information regarding the main presenting symptoms, cancer diagnosis, demographic characteristics, and route of diagnosis for each patient based on primary care records.

Definition and identification of cases

The nature of cancer diagnosis (incidental or non-incidental), was ascertained by examination of the free-text information included in the presenting symptoms data field (answering the audit question "what were the main presenting symptom(s) [of the patient]?").

Tumours were deemed to have been diagnosed incidentally if the incidental nature of diagnosis was explicitly recorded by the participating healthcare professional (indicated by phrases including "accidental finding"; "chance finding"; "incidental"; "opportunistic"), or if the clinical circumstances described were consistent with incidental identification based on clinical knowledge (GL and GPR) and prior literature [5,16,17]. Cases were initially identified by MMK, and subsequently reviewed and validated by GL and GPR; disagreements were resolved by discussion.

Information was available on the patient's sex and age group, and cancer site (categorised as Bladder, Brain, Cervical, Colorectal, Endometrial, Gallbladder, Leukaemia (of any type), Laryngeal, Liver, Lung, Lymphoma, Melanoma, Mesothelioma, Multiple Myeloma, Oesophageal, Oropharyngeal, Ovarian, Pancreatic, Renal, Sarcoma (of any type), Small Intestine, Stomach, Testicular, Thyroid and Vulval) [14]. Patients diagnosed with prostate cancer were excluded *a priori*, given the difficulties in reliably distinguishing reasons for Prostate Specific Antigen testing [18]. Patients with screen-detected breast, colorectal, and cervical cancer, and those diagnosed through surveillance for pre-malignant or high-risk conditions were also excluded. Therefore, the study population comprised 13,810 patients aged 15 or older with sufficient information to determine incidental/non-incidental status, and complete information on cancer diagnosis, age group, and sex (see Supplementary Figure 1 for sample derivation).

Data analysis

Firstly, we compared the demographic and clinical characteristics of incidentally and non-incidentally diagnosed patients. Logistic regression was used to calculate crude and adjusted odds ratios of incidental diagnosis by sex, age group, and cancer site. We also examined the cancer site case-mix ('cancer site signature') of the incidentally diagnosed population, i.e. the relatively frequency of each cancer site among incidentally diagnosed patients. Colorectal cancer was used as the reference category for cancer site, as the most common non-sex specific cancer in our population. All statistical analyses were conducted in STATA SE v.15 (StataCorp, College Station, TX, USA).

For peer review only

Subsequently, we identified common clinical scenarios leading to incidental diagnosis based on a subgroup of patients with relevant information (n=345, 66% of all incidental diagnoses). These findings were synthesised narratively.

Sensitivity analysis

We performed sensitivity analyses expanding the definition of incidental diagnosis of cancer to include an additional 272 patients without registered presenting symptoms, and/or with abnormal clinical findings to the audit question "what were the main presenting symptom [of the patient]?".

Ethical approval

Ethical approval was not required given the anonymous nature of these data.

Patient and public involvement

Patients and members of the public were not involved in the design of this study.

For peer review only

RESULTS

Incidentally diagnosed cancer patients

A total of 520/13,810 (4%) patients aged 15+ years were diagnosed incidentally with one of 25 cancer sites (other than prostate cancer). Men were more likely to be diagnosed incidentally than women (5% of men vs 3% of women), although there was no evidence to support this after adjustment for age and cancer site (see Table 1). The odds of being diagnosed incidentally with cancer generally increased with age (joint Wald test p-value = <0.001).

Crude and adjusted odds ratios indicated substantial variation in the odds of incidental diagnosis between cancer sites (see Figure 1 and Table 1). Almost a quarter (23%) of leukaemia patients and over a tenth (13%) of all renal cancer patients were diagnosed incidentally. More than a tenth of patients with thyroid (12%) and liver cancer (11%) were also diagnosed incidentally. In contrast, less than 1% of patients with endometrial, testicular, breast, sarcoma, brain, oesophageal and cervical cancers were diagnosed incidentally.

For peer review only

Table 1 Characteristics of incidental cancer patients versus non-incidental cancer patients, and crude/adjusted odds ratios of incidental status (n=13,810)

	Total	Incidental		Crude	Adjusted[a]
	N	n	% (95% CI)	OR (95% CI)	OR (95% CI)
Total	13810	520	4% (3–4%)	-	-
**Sex**				**0.001**[b]	0.204[b]
Men	5839	278	5% (4–5%)	Ref.	Ref.
Women	7971	242	3% (3–3%)	0.63 (0.53–0.75)	0.88 (0.72–1.07)
**Age group**				**<0.001**[b]	**<0.001**[b]
15–49 years	2072	31	1% (1–2%)	0.40 (0.27–0.59)	0.39 (0.26–0.60)
50–59 years	2050	65	3% (2–4%)	0.86 (0.63–1.17)	0.88 (0.64–1.21)
60–69 years	3181	117	4% (3–4%)	Ref.	Ref.
70–79 years	3656	170	5% (4–5%)	1.28 (1.00–1.62)	1.28 (1.00–1.64)
80+ years	2851	137	5% (4–6%)	1.32 (1.03–1.70)	1.45 (1.12–1.89)
**Cancer site**				**<0.001**[b]	**<0.001**[b]
Leukaemia*	450	103	23% (19–27%)	10.49 (7.55–14.58)	11.84 (8.49–16.51)
Renal	356	46	13% (10–17%)	5.25 (3.53–7.78)	5.60 (3.77–8.33)
Thyroid	110	13	12% (7–19%)	4.74 (2.53–8.88)	7.25 (3.80–13.82)
Liver	103	11	11% (6–18%)	4.23 (2.16–8.27)	4.42 (2.24–8.68)
Myeloma	228	20	9% (6–13%)	3.40 (2.02–5.72)	3.39 (2.01–5.70)
Gallbladder	68	5	7% (3–16%)	2.81 (1.09–7.20)	2.96 (1.15–7.62)
Mesothelioma	75	4	5% (2–13%)	1.99 (0.71–5.61)	1.88 (0.66–5.31)
Lymphoma	698	33	5% (3–7%)	1.75 (1.14–2.69)	2.10 (1.37–3.23)
Vulval	73	3	4% (1–11%)	1.51 (0.46–4.94)	1.70 (0.52–5.60)
Lung	1875	77	4% (3–5%)	1.51 (1.08–2.12)	1.49 (1.07–2.09)
Melanoma	834	30	4% (3–5%)	1.32 (0.85–2.05)	1.69 (1.09–2.64)
Bladder	842	28	3% (2–5%)	1.22 (0.78–1.91)	1.16 (0.74–1.82)
Colorectal	2399	66	3% (2–3%)	Ref.	Ref.
Stomach	302	8	3% (1–5%)	0.96 (0.46–2.02)	0.94 (0.45–1.99)
Ovarian	394	10	3% (1–5%)	0.92 (0.47–1.81)	1.11 (0.56–2.20)
Laryngeal	121	3	2% (1–7%)	0.90 (0.28–2.90)	0.96 (0.30–3.12)
Oropharyngeal	213	5	2% (1–5%)	0.85 (0.34–2.13)	1.05 (0.42–2.64)
Small Intestine	53	1	2% (0.3–10%)	0.68 (0.09–4.99)	0.72 (0.10–5.28)
Pancreatic	370	6	2% (1–3%)	0.58 (0.25–1.35)	0.59 (0.26–1.38)
Endometrial	410	6	1% (1–3%)	0.52 (0.23–1.22)	0.61 (0.26–1.43)
Testicular	149	2	1% (0.4–5%)	0.48 (0.12–1.98)	1.05 (0.25–4.43)
Breast	2675	34	1% (1–2%)	0.46 (0.30–0.69)	0.59 (0.38–0.91)
Sarcoma*	106	1	0.9% (0.2–5%)	0.34 (0.05–2.45)	0.43 (0.06–3.14)
Brain	215	2	0.9% (0.3–3%)	0.33 (0.08–1.36)	0.37 (0.09–1.54)
Oesophageal	566	3	0.5% (0.2–2%)	0.19 (0.06–0.60)	0.19 (0.06–0.60)
Cervical	125	0	0% (0–3%)	N/A	N/A

[a] adjusted for sex, age group, and cancer site
[b] joint Wald test p-value
*No information was available on leukaemia or sarcoma type.

For peer review only

Among the 520 incidentally diagnosed patients, a fifth (20%, 95% CI: 17–23%) were diagnosed with leukaemia, while other common cancer sites included lung (15%, 12–18%), colorectal (13%, 10–16%), and renal cancers (9%, 7–12%) (see Figure 2 and Supplementary Table 1). There were 9 other cancer sites represented amongst the incidentally diagnosed cancer patient population with 10 or more patients each.

Sensitivity analyses (using a broader definition of incidental diagnosis) identified a further 272 cases, increasing the overall estimate of incidental diagnosis to 6% (see Supplementary Table 2 and Supplementary Figure 2). There was weak evidence to support greater odds of incidental diagnosis among men versus women (adjusted OR (95% CI): 0.84 (0.71–1.00)), with otherwise similar patterns of variation by age group and cancer site as in the main analysis.

Routes to incidental cancer diagnosis

We identified several clinical scenarios preceding an incidental diagnosis of cancer based on information available for 345 patients (66% of all incidentally diagnosed patients). These are outlined in Table 2 and discussed in further detail below.

Many patients received an incidental cancer diagnosis as a result of a clinical encounter for a pre-existing chronic disease in primary or secondary care. This included routine blood or urine testing, as part of chronic disease (or related risk factor) management and monitoring, which revealed abnormalities that led to the diagnosis of unsuspected cancer. Some patients were diagnosed following blood or imaging investigations before/after elective surgery for unrelated indications with a small number of patients where tumours were identified during surgery. A small number of patients were diagnosed after blood or imaging investigations conducted as part of follow up for a pre-existing cancer (e.g. scans to ascertain stage at diagnosis of prostate cancer led to the diagnosis of a renal cancer).

Other cancer patients were diagnosed following the investigation of unrelated acute conditions or presenting symptom(s) unlikely to be related to the subsequent cancer diagnosis. Several of these cases were being investigated for another suspected cancer (e.g. a CT scan for a suspected pelvic cancer leading to the diagnosis of colorectal cancer) but in others the diagnosis was more serendipitous (e.g. breast lump found on examination for chest infection).

Table 2 Clinical scenarios preceding the incidental diagnosis of cancer

Clinical scenario	Description and examples
Monitoring or managing pre-existing chronic morbidity	Blood or imaging investigations as part of monitoring or management of a chronic morbidity<br>E.g. haematuria on dipstick urine testing [for diabetes] led to diagnosis of bladder cancer<br>E.g. annual blood tests for hypertension led to diagnosis of leukaemia
Before/after surgery	Blood or imaging investigations conducted before or after surgery, or more rarely, tumours identified during elective surgery for unrelated condition<br>E.g. pre-operative chest x-ray leading to diagnosis of lung cancer<br>E.g. microscopic haematuria noted pre-cataract operation leading to diagnosis of a urological cancer
Follow up of a pre-existing cancer	Blood or imaging investigations conducted as part of follow up for a pre-existing cancer<br>E.g. scans to ascertain stage at diagnosis of prostate cancer leading to the diagnosis of a urological cancer
Investigation of unrelated acute or new condition or symptoms	Blood or imaging investigations for a new symptom or otherwise acute condition<br>E.g. an abdominal ultrasound scan for dyspepsia leading to the diagnosis of a urological cancer<br>E.g. abnormal result or irregular mole noted during health check

For peer review only

For peer review only

DISCUSSION

Principal findings

Around 1 in 25 cancer patients in our study population were diagnosed incidentally, with a preponderance among older patients, and patients with leukaemia, renal cancer, thyroid cancer, liver cancer, and multiple myeloma. Several clinical scenarios preceded incidental diagnosis including healthcare encounters due to previously known conditions and the investigation of acute or new conditions unrelated to cancer.

Strengths and limitations

Our study is based on a cohort of cancer patients (diagnosed 2009–10) and is therefore limited by temporality of the data. However, thus far there have been no subsequent population-based data collections that could enable the detailed examination of the context of presentation in patients subsequently diagnosed with cancer in England. Information on incidental status at diagnosis is not routinely recorded as part of cancer registration data, nor coded as such in administrative databases or patient experience surveys. A strength of our study is that it provides unique evidence about this less well documented diagnostic pathway of cancer, among a large and representative incident cohort characterised by healthcare professionals.

Nevertheless, interpretation of the findings should be mindful of the secondary nature of our analysis. Information on symptoms (or their absence) was based on those recorded in primary care; patients found to be asymptomatic by auditors may have had symptoms that were either not declared during the consultation, or else not recorded in their records [19,20]. In order to reduce the risk of the resulting bias on analyses, our definition of incidentally diagnosed cancer was deliberately conservative, designed to maximise specificity and reduce the likelihood of patients being mistakenly identified as incidental diagnoses. However, this may have led to the under-estimation of cases; our sensitivity analysis (based on a less conservative definition) indicates that an additional 2% of the study population may have been incidentally diagnosed (Supplementary Table 2). Although the true estimates of incidental diagnosis may be higher than those reported, this is unlikely to have biased patterns of variation by cancer site and patient characteristics.

Comparison with existing literature

Literature examining incidentally diagnosed cancer is limited, although some evidence may be gleaned from studies on incidental findings detected in the context of research studies. Estimates of clinically important incidental findings (including cancer but also other diseases) vary substantially depending on imaging field (whole body, or specific organ) and modality however, and participants of research studies are unlikely to be representative of the general population [21,22].

Though we were unable to examine potential overdiagnosis, we identified notable proportions of incidentally diagnosed patients with thyroid and renal cancer, and melanoma patients. This is consistent with prior evidence indicating potential overdiagnosis of these cancers [23–26]. A few studies have examined clinical scenarios that result in incidental diagnosis of individual cancer sites such as melanoma, lung cancer, and renal cancer [17,27–29]. A study examining self-reported symptoms of haematological cancer patients found that a third of patients did not report any symptoms before diagnosis, with chronic lymphocytic leukaemia patients being particularly prone to being diagnosed incidentally, for example through blood tests at routine healthcare encounters [30].

For peer review only

Our findings are in agreement with these studies, but additionally suggest that incidental diagnosis occurs across a range of common and rarer cancers.

Implications

Currently, there is sparse evidence regarding the prevalence or incidence of incidentally diagnosed cancer, likely due to the challenges in identifying such cases using large administrative healthcare data. Using unique data from an audit initiative, we were able to identify several clinical scenarios resulting in incidental diagnosis of cancer. This study provides important epidemiological evidence quantifying the frequency of such cases, and characterising the different mechanisms that can lead to an incidental cancer diagnosis.

Our findings indicate that a substantial proportion of cancer patients are diagnosed with cancer incidentally, without having presented with symptoms related to the subsequent diagnosis. An incidental cancer diagnosis could represent fortuitous early diagnosis of an invasive tumour, and therefore be of clinical benefit for a proportion of patients. However it could also represent overdiagnosis, which could lead to considerable psychological morbidity and unnecessary treatment.

The frequency of incidental diagnosis, and the relative frequency of the scenarios preceding incidental diagnosis are likely to be affected by system level factors such as approaches to chronic disease monitoring, incentives and thresholds for investigation, availability of imaging services, and rates of elective surgery [31,32]. Given increasing levels of multi-morbidity and an ageing population, there is progressively greater use of blood-based testing and imaging studies, which could lead to a greater proportion of patients being diagnosed incidentally particularly for certain cancer types such as leukaemia [10]. Relatedly, incidental diagnosis of cancer occurred during investigation or follow up of a pre-existing (unrelated) tumour in a small number of patients. As the survival of patients with cancer continues to improve, this could also become a more prevalent route to incidental diagnosis [33]. Further examination of incidentally diagnosed cancer among more contemporary populations, and incidence trends of such diagnoses would be helpful in this regard, particularly given that it may represent overdiagnosis.

Conclusions

In conclusion, we have provided evidence about the frequency and common scenarios leading to incidental diagnosis of cancer. Our findings indicate that this is likely to affect around one in 25 cancer patients and calls for further research establishing the prognostic, psychosocial and economic implications of incidentally diagnosed cancer.

For peer review only

REFERENCES

1 Elliss-Brookes L, McPhail S, Ives A, *et al.* Routes to diagnosis for cancer - determining the patient journey using multiple routine data sets. *Br J Cancer* 2012;**107**:1220–6. doi:10.1038/bjc.2012.408

2 Jensen H, Tørring ML, Olesen F, *et al.* Cancer suspicion in general practice, urgent referral and time to diagnosis: a population-based GP survey and registry study. *BMC Cancer* 2014;**14**:636. doi:10.1186/1471-2407-14-636

3 Lumbreras B, Donat L, Hernández-Aguado I. Incidental findings in imaging diagnostic tests: a systematic review. *Br J Radiol* 2010;**83**:276–89. doi:10.1259/bjr/98067945

4 Kroczek EK, Wieners G, Steffen I, *et al.* Non-traumatic incidental findings in patients undergoing whole-body computed tomography at initial emergency admission. *Emerg Med J* 2017;:emermed-2016-205722. doi:10.1136/emermed-2016-205722

5 O'Sullivan JW, Muntinga T, Grigg S, *et al.* Prevalence and outcomes of incidental imaging findings: umbrella review. *BMJ* 2018;:k2387. doi:10.1136/bmj.k2387

6 Maskell G. Think before you scan. *Bmj* 2018;**3754**:k3754. doi:10.1136/bmj.k3754

7 NHS. NHS Health Check. 2017.http://www.healthcheck.nhs.uk/

8 NHS Digital. Quality and Outcomes Framework. Prim. Care. 2016.http://content.digital.nhs.uk/QOF

9 Treadwell J, McCartney M. Overdiagnosis and overtreatment: generalists--it's time for a grassroots revolution. *Br J Gen Pract* 2016;**66**:116–7. doi:10.3399/bjgp16X683881

10 O'Sullivan JW, Stevens S, Hobbs FDR, *et al.* Temporal trends in use of tests in UK primary care, 2000-15: retrospective analysis of 250 million tests. *Bmj* 2018;:k4666. doi:10.1136/bmj.k4666

11 Esserman LJ, Thompson IM, Reid B, *et al.* Addressing overdiagnosis and overtreatment in cancer: A prescription for change. *Lancet Oncol* 2014;**15**:e234–42. doi:10.1016/S1470-2045(13)70598-9

12 Jenniskens K, de Groot JAH, Reitsma JB, *et al.* Overdiagnosis across medical disciplines: a scoping review. *BMJ Open* 2017;**7**:e018448. doi:10.1136/bmjopen-2017-018448

13 Davies L, Petitti DB, Martin L, *et al.* Defining, Estimating, and Communicating Overdiagnosis in Cancer Screening. *Ann Intern Med* 2018;**169**:36. doi:10.7326/M18-0694

14 Rubin GP, McPhail S, Elliot K, *et al.* National Audit of Cancer Diagnosis in Primary Care. London: 2011. http://www.rcgp.org.uk/policy/rcgp-policy-areas/national-audit-of-cancer-diagnosis-in-primary-care.aspx

15 Lyratzopoulos G, Abel GA, McPhail S, *et al.* Gender inequalities in the promptness of diagnosis of bladder and renal cancer after symptomatic presentation: evidence from secondary analysis of an English primary care audit survey. *BMJ Open* 2013;**3**:e002861. doi:10.1136/bmjopen-2013-002861

16 Davies L, Ouellette M, Hunter M, *et al.* The increasing incidence of small thyroid cancers: Where are the cases coming from? *Laryngoscope* 2010;**120**:2446–51. doi:10.1002/lary.21076

17 Kocher F, Lunger F, Seeber A, *et al.* Incidental Diagnosis of Asymptomatic Non-Small-Cell Lung Cancer: A Registry-Based Analysis. *Clin Lung Cancer* 2016;**17**:62–7. doi:10.1016/j.cllc.2015.08.006

For peer review only

18 NICE. Prostate cancer : diagnosis and management (CG175). Published Online First: 2014.https://www.nice.org.uk/guidance/cg175

19 Larsen MB, Hansen RP, Sokolowski I, *et al.* Agreement between patient-reported and doctor-reported patient intervals and date of first symptom presentation in cancer diagnosis – A population-based questionnaire study. *Cancer Epidemiol* 2014;**38**:100–5. doi:10.1016/j.canep.2013.10.006

20 Leiva A, Esteva M, Llobera J, *et al.* Time to diagnosis and stage of symptomatic colorectal cancer determined by three different sources of information: A population based retrospective study. *Cancer Epidemiol* 2017;**47**:48–55. doi:10.1016/j.canep.2016.10.021

21 The Royal College of Radiologists. Management of Incidental Findings Detected During Research Imaging. 2011. papers3://publication/uuid/627D4627-FE73-472B-BD26-9FD0F38FC547

22 Booth TC, Jackson A, Wardlaw JM, *et al.* Incidental findings found in 'healthy' volunteers during imaging performed for research: Current legal and ethical implications. *Br J Radiol* 2010;**83**:456–65. doi:10.1259/bjr/15877332

23 Weyers W. The 'epidemic' of melanoma between under- and overdiagnosis. *J Cutan Pathol* 2012;**39**:9–16. doi:10.1111/j.1600-0560.2011.01831.x

24 Welch HG, Black WC. Overdiagnosis in cancer. *J Natl Cancer Inst* 2010;**102**:605–13. doi:10.1093/jnci/djq099

25 Moynihan R, Doust J, Henry D. Preventing overdiagnosis: how to stop harming the healthy. *Bmj* 2012;**344**:e3502–e3502. doi:10.1136/bmj.e3502

26 Ahn HS, Kim HJ, Welch HG. Korea's Thyroid-Cancer "Epidemic" — Screening and Overdiagnosis. *N Engl J Med* 2014;**371**:1765–7. doi:10.1056/NEJMp1409841

27 Cufari ME, Proli C, Phull M, *et al.* Increasing incidence of non-smoking lung cancer: presentation of patients with early disease to a tertiary institution in the UK. *Lung Cancer* 2016;**91**:S17–8. doi:10.1016/S0169-5002(16)30066-6

28 Avilés-Izquierdo JA, Molina-López I, Rodríguez-Lomba E, *et al.* Who detects melanoma? Impact of detection patterns on characteristics and prognosis of patients with melanoma. *J Am Acad Dermatol* 2016;:1–8. doi:10.1016/j.jaad.2016.07.009

29 Hofbauer SL, de Martino M, Seemann C, *et al.* Associations between presenting symptoms, clinicopathological parameters, and prognosis in a contemporary series of patients with renal cell carcinoma. *Korean J Urol* 2014;**55**:505–10. doi:10.4111/kju.2014.55.8.505

30 Howell DA, Smith AG, Jack A, *et al.* Time-to-diagnosis and symptoms of myeloma, lymphomas and leukaemias: a report from the Haematological Malignancy Research Network. *BMC Blood Disord* 2013;**13**:9. doi:10.1186/2052-1839-13-9

31 Pollack CE, Soulos PR, Herrin J, *et al.* The Impact of Social Contagion on Physician Adoption of Advanced Imaging Tests in Breast Cancer. *J Natl Cancer Inst* 2017;**109**:1–8. doi:10.1093/jnci/djw330

32 Barraclough K. New NICE guidance on referral for cancer. *BMJ* 2015;**351**:h3640. doi:10.1136/bmj.h3640

33 Murphy CC, Gerber DE, Pruitt SL. Prevalence of Prior Cancer Among Persons Newly Diagnosed With Cancer. *JAMA Oncol* 2017;**75390**:1–4. doi:10.1001/jamaoncol.2017.3605

For peer review only

For peer review only

Additional information

Acknowledgements

We are grateful to all general practitioners and healthcare professionals that were involved in the collection and submission of anonymous data to the audit, and to the respective Cancer Networks, the Royal College of General Practitioners, the former National Cancer Action Team and the former National Clinical Intelligence Network (NCIN) of Public Health England (PHE) for supporting the audit.

Competing interests

The authors have declared no competing interests.

Funding statement

This work was supported by the UK Department of Health as part of the programme of the Policy Research Unit in Cancer Awareness, Screening and Early Diagnosis [grant number no. 106/0001]. The Policy Research Unit in Cancer Awareness, Screening, and Early Diagnosis is a collaboration between researchers from seven institutions (Queen Mary University of London, University College London, King's College London, London School of Hygiene and Tropical Medicine, Hull York Medical School, Durham University and Peninsula Medical School/University of Exeter). GL is supported by Cancer Research UK Clinician Advanced Scientist Fellowship [grant number: C18081/A18180]. The views expressed are those of the authors and not necessarily those of the Department of Health or Cancer Research UK. The funders of the study had no role in the study design, data analysis, data interpretation, or writing of the report. The corresponding author had full access to all the data in the study and had final responsibility for the decision to submit for publication.

Authorship contribution

MMK, GPR, and GL conceived the study. MMK conducted all statistical analyses with assistance from GL. MMK wrote the first draft of the manuscript, and prepared the tables and figures, supervised by GL. MMK, GPR, SMcP, and GL contributed to the interpretation of the results, revised the manuscript and approved the final version of the manuscript.

Data sharing agreement

The data used for our analysis is available from the National Cancer Registration and Analysis Service. Enquiries for data access can be made to Public Health England's Office for Data Release (odr@phe.gov.uk).

Figure/Table legends

Figure 1 Odds ratios of incidental versus symptomatic diagnosis of cancer by cancer site (n=13,810; reference group: colorectal cancer). NB there is no odds ratio for cervical cancer as there were no incidentally diagnosed cases of cervical cancer.

Figure 2 Commonly diagnosed cancer sites among the incidental cancer patient population; see Supplementary Table 1 for frequencies

Table 1 Characteristics of incidental cancer patients versus non-incidental cancer patients, and crude/adjusted odds ratios of incidental status (n=13,810)

Table 2 Clinical scenarios preceding the incidental diagnosis of cancer

For peer review only

For peer review only

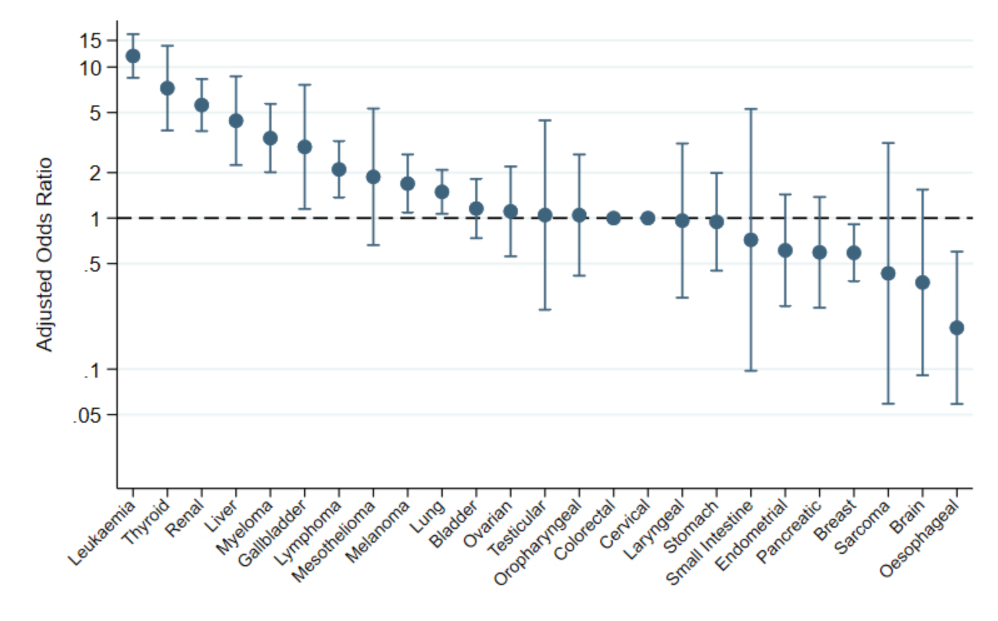

Figure 1: Odds ratios of incidental versus symptomatic diagnosis of cancer by cancer site (n=13,810; reference group: colorectal cancer). NB there is no odds ratio for cervical cancer as there were no incidentally diagnosed cases of cervical cancer.

282x176mm (72 x 72 DPI)

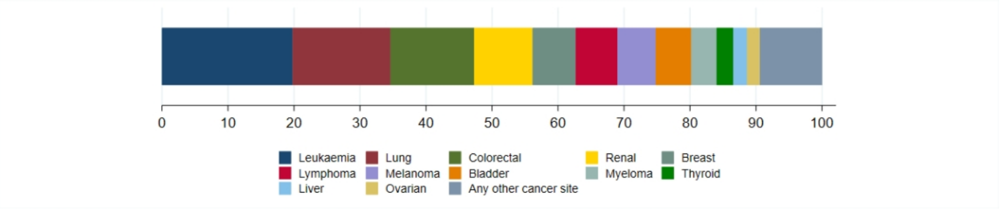

Figure 2: Commonly diagnosed cancer sites among the incidental cancer patient population; see Supplementary Table 1 for frequencies

423x88mm (72 x 72 DPI)

Supplementary information for "*Incidentally diagnosed cancer and commonly preceding clinical scenarios: a cross-sectional descriptive analysis of English audit data*" Koo et al., 2019

Table of contents

- Supplementary Figure 1: Flow chart describing sample derivation for main analysis
- Supplementary Table 1: Cancer site case-mix of incidentally diagnosed cancer patients
- *Sensitivity analysis using a broader definition of incidental diagnosis*
    - Supplementary Table 2: Characteristics of incidental cancer patients versus non-incidental cancer patients, and crude/adjusted odds ratios of incidental status (n=14,082)
    - Supplementary Figure 2: Odds ratios of incidental versus symptomatic diagnosis of cancer by cancer site (n=14,082; reference group: colorectal cancer)

For peer review only

Supplementary Figure 1: Flow chart describing sample derivation for main analysis

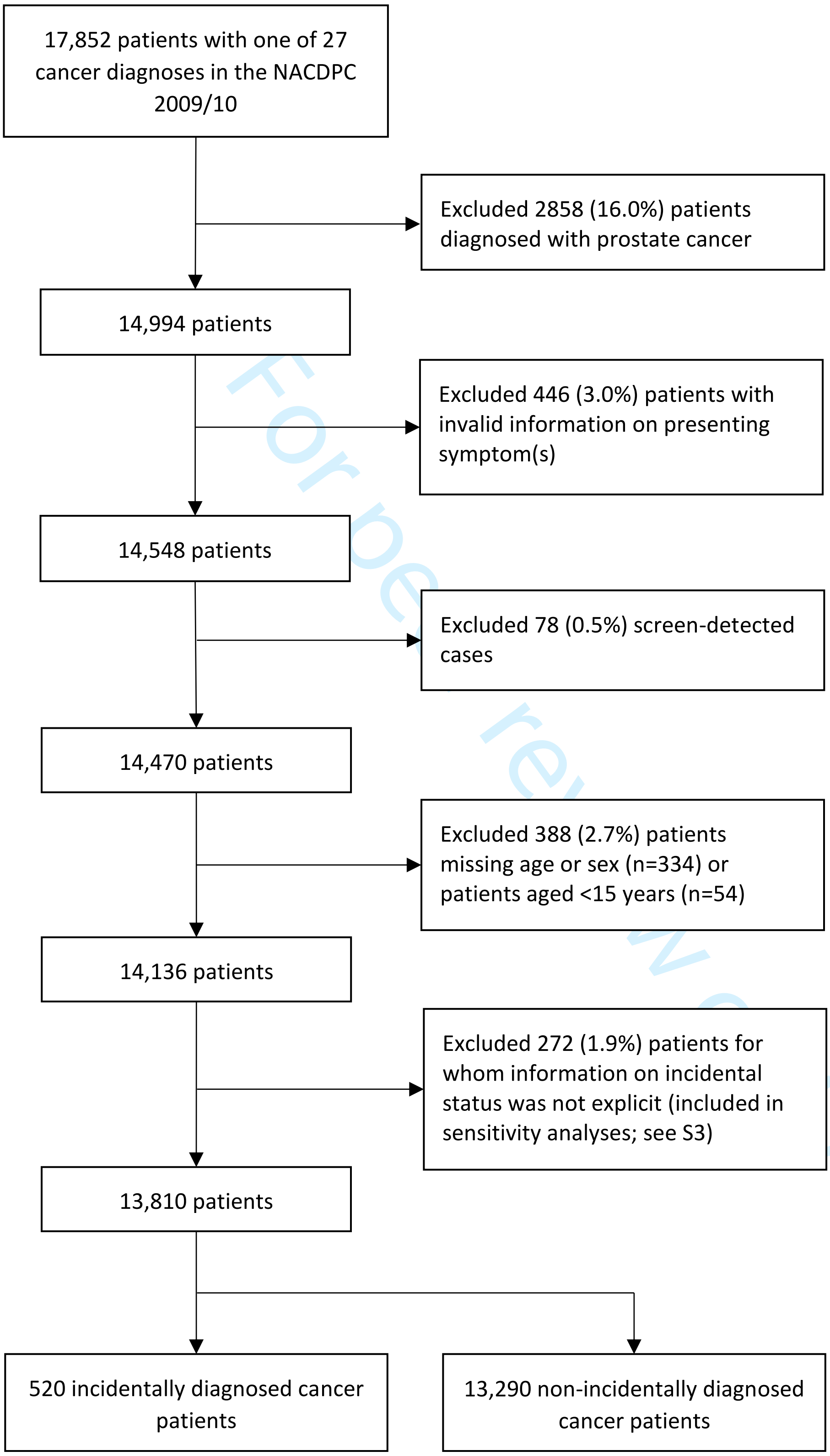

For peer review only - http://bmjopen.bmj.com/site/about/guidelines.xhtml

Supplementary files for Koo et al., 2019

Supplementary Table 1: Cancer site case-mix of incidentally diagnosed cancer patients

Cancer	N	% (95% CI)†
Leukaemia*	103	20% (17–23%)
Lung	77	15% (12–18%)
Colorectal	66	13% (10–16%)
Renal	46	9% (7–12%)
Breast	34	7% (5–9%)
Lymphoma	33	6% (5–9%)
Melanoma	30	6% (4–8%)
Bladder	28	5% (4–8%)
Myeloma	20	4% (3–6%)
Thyroid	13	3% (1–4%)
Liver	11	2.1% (1.2–3.7%)
Ovarian	10	1.9% (1.0–3.5%)
Stomach	8	1.5% (0.8–3.0%)
Endometrial	6	1.2% (0.5–2.5%)
Pancreatic	6	1.2% (0.5–2.5%)
Gallbladder	5	1.0% (0.4–2.2%)
Oropharyngeal	5	1.0% (0.4–2.2%)
Mesothelioma	4	0.8% (0.3–2.0%)
Laryngeal	3	0.6% (0.2–1.7%)
Oesophageal	3	0.6% (0.2–1.7%)
Vulval	3	0.6% (0.2–1.7%)
Brain	2	0.4% (0.1–1.4%)
Testicular	2	0.4% (0.1–1.4%)
Sarcoma*	1	0.2% (0.03–1.1%)
Small Intestine	1	0.2% (0.03–1.1%)
Total	520	100%

† Proportion of patients with each cancer site, of the total incidentally diagnosed population (n=520)
*No information was available on leukaemia or sarcoma type.

For peer review only

Supplementary files for Koo et al., 2019

Supplementary Table 2: Characteristics of incidental cancer patients versus non-incidental cancer patients, and crude/adjusted odds ratios of incidental status (n=14,082)

	Total	Incidental		Crude	Adjusted[a]
	N	n	% (95% CI)	OR (95% CI)	OR (95% CI)
Total	14082	792	6% (5–6%)	-	-
**Sex**				**0.001[b]**	**0.045[b]**
Men	5983	422	7% (6–8%)	Ref.	Ref.
Women	8099	370	5% (4–5%)	0.63 (0.55–0.73)	0.84 (0.71–1.00)
**Age group**				**<0.001[b]**	**<0.001[b]**
15–49 years	2089	48	2% (2–3%)	0.36 (0.26–0.50)	0.36 (0.26–0.51)
50–59 years	2080	95	5% (4–6%)	0.73 (0.57–0.94)	0.75 (0.58–0.98)
60–69 years	3264	200	6% (5–7%)	Ref.	Ref.
70–79 years	3739	253	7% (6–8%)	1.11 (0.92–1.35)	1.08 (0.89–1.33)
80+ years	2910	196	7% (6–8%)	1.11 (0.90–1.36)	1.17 (0.94–1.45)
**Cancer site**				**<0.001[b]**	**<0.001[b]**
Leukaemia*	511	164	32% (28–36%)	11.25 (8.55–14.81)	12.48 (9.46–16.48)
Liver	116	24	21% (14–29%)	6.21 (3.79–10.16)	6.28 (3.82–10.32)
Renal	373	63	17% (13–21%)	4.84 (3.45–6.78)	5.12 (3.64–7.19)
Thyroid	113	16	14% (9–22%)	3.93 (2.23–6.92)	5.93 (3.31–10.60)
Myeloma	241	33	14% (10–19%)	3.78 (2.48–5.74)	3.74 (2.46–5.69)
Gallbladder	68	5	7% (3–16%)	1.89 (0.74–4.80)	2.00 (0.78–5.09)
Bladder	869	55	6% (5–8%)	1.61 (1.15–2.26)	1.52 (1.08–2.14)
Lung	1913	115	6% (5–7%)	1.52 (1.15–2.01)	1.50 (1.14–1.97)
Lymphoma	704	39	6% (4–7%)	1.40 (0.95–2.04)	1.64 (1.12–2.41)
Mesothelioma	75	4	5% (2–13%)	1.34 (0.48–3.75)	1.23 (0.44–3.45)
Melanoma	839	35	4% (3–6%)	1.04 (0.70–1.54)	1.30 (0.88–1.94)
Vulval	73	3	4% (1–11%)	1.02 (0.32–3.30)	1.19 (0.37–3.87)
Colorectal	2431	98	4% (3–5%)	Ref.	Ref.
Ovarian	398	14	4% (2–6%)	0.87 (0.49–1.54)	1.05 (0.59–1.88)
Stomach	303	9	3% (2–6%)	0.73 (0.36–1.46)	0.72 (0.36–1.44)
Breast	2717	76	3% (2–3%)	0.69 (0.51–0.93)	0.91 (0.66–1.25)
Pancreatic	374	10	3% (1–5%)	0.65 (0.34–1.27)	0.66 (0.34–1.28)
Laryngeal	121	3	2% (1–7%)	0.61 (0.19–1.94)	0.62 (0.19–1.98)
Oropharyngeal	213	5	2% (1–5%)	0.57 (0.23–1.42)	0.68 (0.27–1.69)
Endometrial	413	9	2% (1–4%)	0.53 (0.27–1.06)	0.62 (0.31–1.24)
Small Intestine	53	1	2% (–10%)	0.46 (0.06–3.35)	0.48 (0.07–3.55)
Sarcoma*	107	2	2% (1–7%)	0.45 (0.11–1.86)	0.57 (0.14–2.36)
Testicular	149	2	1% (–5%)	0.32 (0.08–1.33)	0.66 (0.16–2.76)
Brain	215	2	1% (–3%)	0.22 (0.05–0.91)	0.25 (0.06–1.02)
Cervical	126	1	1% (–4%)	0.19 (0.03–1.38)	0.29 (0.04–2.09)
Oesophageal	567	4	1% (–2%)	0.17 (0.06–0.46)	0.17 (0.06–0.45)

[a] adjusted for sex, age group, and cancer site
[b] joint Wald test p-value
*No information was available on leukaemia or sarcoma type.

For peer review only

Supplementary Figure 2: Odds ratios of incidental versus symptomatic diagnosis of cancer by cancer site (n=14,082; reference group: colorectal cancer)

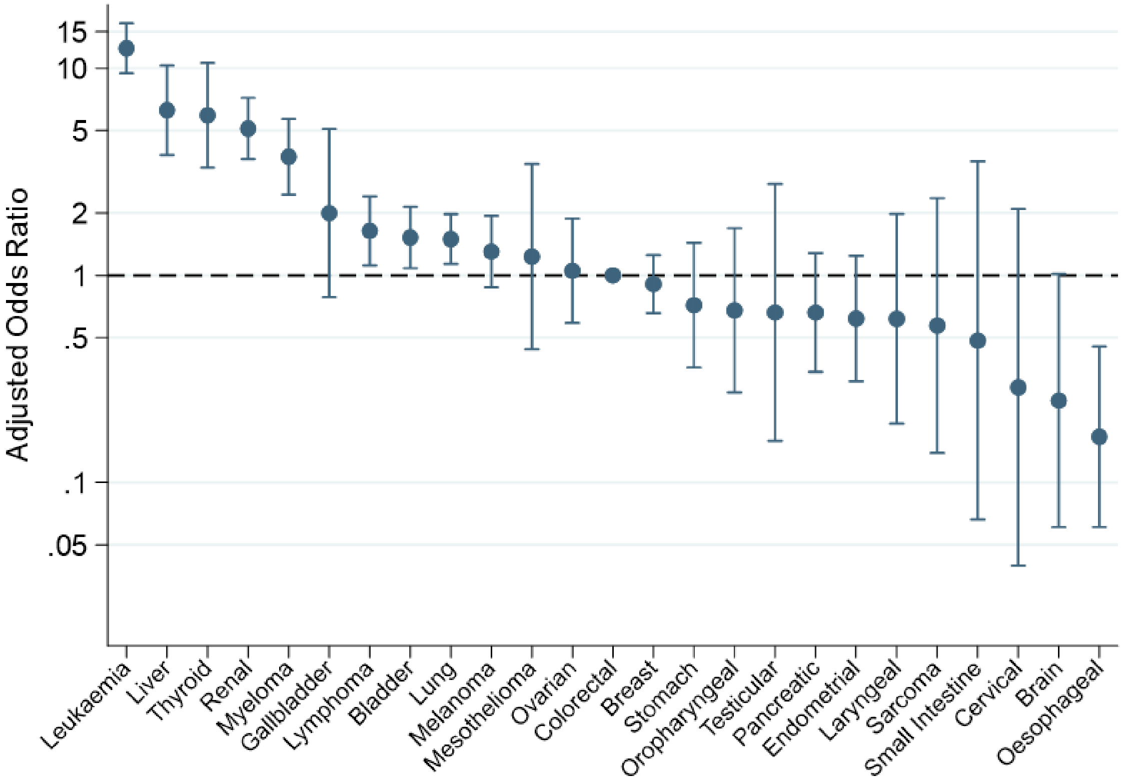

STROBE Statement—Checklist of items that should be included in reports of ***cross-sectional studies***

NB we have added bold text to the "Recommendation" column indicating the presence of each item recommended by the STROBE statement in the submitted BMJ Open manuscript ID bmjopen-2018-028362

	Item No	Recommendation
**Title and abstract**	1	(*a*) Indicate the study's design with a commonly used term in the title or the abstract **See title, first page**
		(*b*) Provide in the abstract an informative and balanced summary of what was done and what was found **See Abstract**
**Introduction**		
Background/rationale	2	Explain the scientific background and rationale for the investigation being reported **See Introduction, first/second paragraphs**
Objectives	3	State specific objectives, including any prespecified hypotheses **See Introduction, last paragraph**
**Methods**		
Study design	4	Present key elements of study design early in the paper **See Methods: Study design and population subsection**
Setting	5	Describe the setting, locations, and relevant dates, including periods of recruitment, exposure, follow-up, and data collection **See Methods: Study design and population subsection**
Participants	6	(*a*) Give the eligibility criteria, and the sources and methods of selection of participants **See Methods: Study design and population subsection**
Variables	7	Clearly define all outcomes, exposures, predictors, potential confounders, and effect modifiers. Give diagnostic criteria, if applicable **See Methods: Definition and identification of cases, and Data analysis subsections**
Data sources/ measurement	8*	For each variable of interest, give sources of data and details of methods of assessment (measurement). Describe comparability of assessment methods if there is more than one group **See Methods: Study design and population; and Methods: Definition and identification of cases subsections**
Bias	9	Describe any efforts to address potential sources of bias **See Methods: Data analysis subsection**
Study size	10	Explain how the study size was arrived at **See Methods: Definition and identification of cases subsection, last sentence, and Figure S1 in Supplementary materials**
Quantitative variables	11	Explain how quantitative variables were handled in the analyses. If applicable, describe which groupings were chosen and why **See Methods: Data analysis subsection**
Statistical methods	12	(*a*) Describe all statistical methods, including those used to control for confounding **See Methods: Data analysis subsection**
		(*b*) Describe any methods used to examine subgroups and interactions **N/A**
		(*c*) Explain how missing data were addressed

For peer review only - http://bmjopen.bmj.com/site/about/guidelines.xhtml

		**See Discussion: Strengths and limitations subsection**
		(*d*) If applicable, describe analytical methods taking account of sampling strategy **N/A**
		(*e*) Describe any sensitivity analyses **See Methods: Sensitivity analysis subsection**
**Results**		
Participants	13*	(a) Report numbers of individuals at each stage of study—eg numbers potentially eligible, examined for eligibility, confirmed eligible, included in the study, completing follow-up, and analysed **See Methods: Definition and identification of cases subsection and Figure S1 in Supplementary materials**
		(b) Give reasons for non-participation at each stage **See Methods: Definition and identification of cases subsection**
		(c) Consider use of a flow diagram **See Figure S1 in Supplementary materials**
Descriptive data	14*	(a) Give characteristics of study participants (eg demographic, clinical, social) and information on exposures and potential confounders **See Results: Incidentally diagnosed cancer patients subsection and Table 1, first 3 columns excluding the left-most column**
		(b) Indicate number of participants with missing data for each variable of interest **N/A**
Outcome data	15*	Report numbers of outcome events or summary measures **See Results: Incidentally diagnosed cancer patients subsection and Table 1, first 3 columns excluding the left-most column**
Main results	16	(*a*) Give unadjusted estimates and, if applicable, confounder-adjusted estimates and their precision (eg, 95% confidence interval). Make clear which confounders were adjusted for and why they were included **See Results: Incidentally diagnosed cancer patients subsection, Table 1, Figure 3, Table S2**
		(*b*) Report category boundaries when continuous variables were categorized **N/A**
		(*c*) If relevant, consider translating estimates of relative risk into absolute risk for a meaningful time period **N/A**
Other analyses	17	Report other analyses done—eg analyses of subgroups and interactions, and sensitivity analyses **See Results: Incidentally diagnosed cancer patients subsection paragraph 3 for details of the sensitivity analysis**
**Discussion**		
Key results	18	Summarise key results with reference to study objectives **See Discussion: Principal findings subsection**
Limitations	19	Discuss limitations of the study, taking into account sources of potential bias or imprecision. Discuss both direction and magnitude of any potential bias **See Discussion: strengths and limitations subsection**
Interpretation	20	Give a cautious overall interpretation of results considering objectives, limitations, multiplicity of analyses, results from similar studies, and other relevant evidence **See Discussion: strengths and limitations and Discussion: comparison with**

For peer review only - http://bmjopen.bmj.com/site/about/guidelines.xhtml

		**existing literature subsections**
Generalisability	21	Discuss the generalisability (external validity) of the study results **See Discussion: strengths and limitations subsection**
**Other information**		
Funding	22	Give the source of funding and the role of the funders for the present study and, if applicable, for the original study on which the present article is based **See Additional information: Funding statement subsection**

*Give information separately for exposed and unexposed groups.

Note: An Explanation and Elaboration article discusses each checklist item and gives methodological background and published examples of transparent reporting. The STROBE checklist is best used in conjunction with this article (freely available on the Web sites of PLoS Medicine at http://www.plosmedicine.org/, Annals of Internal Medicine at http://www.annals.org/, and Epidemiology at http://www.epidem.com/). Information on the STROBE Initiative is available at www.strobe-statement.org.

For peer review only - http://bmjopen.bmj.com/site/about/guidelines.xhtml
